# Children learn ergative case marking in Hindi using statistical preemption and clause-level semantics (intentionality): evidence from acceptability judgment and elicited production studies with children and adults

**DOI:** 10.12688/openreseurope.15611.2

**Published:** 2023-09-13

**Authors:** Ramya Maitreyee, Gaurav Saxena, Bhuvana Narasimhan, Dipti Misra Sharma, Pruthwik Mishra, Rukmini Bhaya Nair, Soumitra Samanta, Ben Ambridge

**Affiliations:** 1 School of Health and Social Care,, University of Essex, Colchester, Essex, CO4 3SQ, UK; 2 School of Psychological Science, University of Bristol, Bristol, BS8 1TU, UK; 3 Department of Linguistics, University of Colorado, Boulder, Boulder, Colorado, 80309, USA; 4 Language Technologies Research Centre, International Institute of Information Technology-Hyderabad, Gachibowli, Hyderabad, 500032, India; 5 School of Languages, Linguistics and Film, Queen Mary University of London, London, E1 4NS, UK; 6 Department of Humanities and Social Sciences, Indian Institute of Technology Delhi, Hauz Khas, New Delhi, 110016, India; 7 Department of Computer Science, Ramakrishna Mission Vivekananda Educational and Research Institute, Belur Math, Howrah, West Bengal, 711202, India; 8 Division of Psychology, Communication and Human Neuroscience, University of Manchester, Manchester, Greater Manchester, M13 9PL, UK; 9 ESRC International Centre for Language and Communicative Development (LuCiD), International, UK

**Keywords:** child language acquisition, preemption, semantics, ergative marking, Hindi

## Abstract

**Background:** A question that lies at the very heart of language acquisition research is how children learn semi-regular systems with exceptions (e.g., the English plural rule that yields *cats, dogs*, etc, with exceptions *feet* and *men*). We investigated this question for Hindi ergative *ne* marking; another semi-regular but exception-filled system. Generally, in the past tense, the subject of two-participant transitive verbs (e.g., *Ram broke the cup*) is marked with *ne*, but there are exceptions. How, then, do children learn when *ne* marking is required, when it is optional, and when it is ungrammatical?

**Methods:** We conducted two studies using (a) acceptability judgment and (b) elicited production methods with children (aged 4-5, 5-6 and 9-10 years) and adults.

**Results:** All age groups showed effects of *statistical preemption*: the greater the frequency with which a particular verb appears with versus without *ne* marking on the subject – relative to other verbs – the greater the extent to which participants (a) accepted and (b) produced *ne* over zero-marked subjects. Both children and adults also showed effects of clause-level semantics, showing greater acceptance of *ne* over zero-marked subjects for intentional than unintentional actions. Some evidence of semantic effects at the level of the verb was observed in the elicited production task for children and the judgment task for adults.

**Conclusions:** participants mainly learn ergative marking on an input-based verb-by-verb basis (i.e., via statistical preemption; verb-level semantics), but are also sensitive to clause-level semantic considerations (i.e., the intentionality of the action). These findings add to a growing body of work which suggests that children learn semi-regular, exception-filled systems using both statistics and semantics.

## Plain language summary

Native language speakers of Hindi often produce sentences such as “ *Ram ne cup todaa* (Ram broke the cup)”. However, not all subjects (i.e., Ram) of verbs in sentences as such receive *ne* marking (e.g., Ram kitab laayaa (Ram brought the book). How, then, do children learn when *ne* marking is required, when it is optional, and when it is ungrammatical? The present study investigated the use of *ne* marking using two tasks: a) asking children (aged 4–5, 5–6 and 9–10 years) and adults to indicate the extent to which sentence they heard was acceptable or unacceptable (“Ram kitab laayaa”, “*Ram-ne kitab laayaa”); b) asking children (aged 4–5, 5–6 years) to produce sentences with verbs where the subject may or may not be marked with a *ne* marker. Both children and adults accepted or produced the *ne* marker with the subject of a verb if they frequently heard the subject of the verb associated with the *ne* marker in the input. Further, children and adults accepted the *ne* marker on subjects when the action was intentional versus unintentional. Thus, these findings suggest that children may learn using partial regularities in the language system based on the input they hear and the properties of the action being performed.

## Introduction

As anyone who has attempted to learn a new language as an adult will know, languages are full of semi-regular patterns, i.e., rules with exceptions. For example, most English nouns form a plural with *-s* (e.g., *cats*, *dogs* etc.), which makes it difficult for children and second-language learners alike to learn exceptions such as *feet* and *men*. At the sentence level, English speakers can say both *The boy moved* and *Somebody moved the boy*, but not both *The boy danced* and *Somebody danced the boy; and again, learning these exceptions proves difficult for first- and second-language learners alike (e.g., Bowerman, 1988; Robenalt & Goldberg, 2016).

The question of exactly how learners acquire these exception-filled generalizations has implications not just for language, but for human cognition more generally (e.g., Pinker, 1999). Are our cognitive categories and processes best thought of as formal rules with memorized exceptions, or as fuzzier, more probabilistic generalizations? This debate has some surprisingly broad implications; for example, how best to design self-driving cars (e.g., Marcus & Davis, 2019). Should such cars be hard-wired with formal rules to follow in particular situations, or simply provided with huge amounts of training data and left to form their own probabilistic generalizations?

Unsurprisingly, given its broad linguistic and non-linguistic implications, the question of how to acquire exception-filled generalizations has inspired a great deal of theoretical debate and empirical research. However, with just a handful of exceptions (e.g., Ambridge *et al*., 2020; Ambridge *et al*., 2022) research on child language has mainly been restricted to English (e.g., Alishahi & Stevenson, 2008; Ambridge *et al*., 2008; Ambridge *et al*., 2009; Ambridge, 2013; Ambridge & Ambridge, 2020; Ambridge & Blything, 2016; Ambridge & Brandt, 2013; Ambridge *et al*., 2011; Ambridge *et al*., 2012a; Ambridge *et al*., 2012b; Ambridge *et al*., 2013; Ambridge *et al*., 2014; Ambridge *et al*., 2015; Ambridge *et al*., 2018; Barak *et al*., 2016; Bidgood *et al*., 2014; Blything *et al*., 2014; Boyd & Goldberg, 2011; Brooks & Zizak, 2002; Brooks *et al*., 1999; Goldberg, 2011; Gropen *et al*., 1991; Harmon & Kapatsinski, 2017; Hsu & Chater, 2010; Irani, 2009; Li & MacWhinney, 1996; Perek & Goldberg, 2017; Perfors *et al*., 2010; Robenalt & Goldberg, 2015; Robenalt & Goldberg, 2016; Stefanowitsch, 2008; Theakston, 2004; Twomey *et al*., 2014; Twomey *et al*., 2016; Wonnacott *et al*., 2008), including two book-length treatments ( Goldberg, 2019; Pinker, 1989).

This focus on English is unfortunate given that most – and quite possibly all – languages exhibit this phenomenon of generalizations with exceptions somewhere in the system. Our goal in the present study is therefore to investigate this problem in a domain i.e., ergative marking in Hindi. The phenomenon is that, for active (i.e., not passive) sentences in the past tense/perfective aspect (i.e., denoting a completed action, but see Singh, 1998), the subject of two-participant transitive sentences (1) – but not single-participant intransitive sentences (2) – is *typically* marked by the clitic marker *ne* ^1^

*(1)*  *Raam **=ne** gilaas=ko toR-aa*
    Ram=Erg glass=Acc break-Msc.Sg.Prf
    Ram broke the glass.
*(2)*  *Raam-0 hãs-aa*
    Ram-Nom laugh-Msc.Sg.Prf
    Ram laughed.

However, some sentences that meet all of the other criteria (i.e., active, past tense/perfective aspect, transitive) still do not show *ne* marking (3); indeed, most native-speaking adults would regard such marking as ungrammatical (4).

*(3)*  *laRkaa-0 raakshas=se Dar-aa*
    boy-Nom demon=Inst fear-Msc.Sg.Prf
    The boy feared the demon.
*(4)*  * *laRke= **ne** raakshas=se Dar-aa*
    boy=Erg demon=Inst fear-Msc.Sg.Prf
    The boy feared the demon.

Complicating the picture still further, in some cases *ne* marking seems to be optional, in that adult native speakers accept both the version with *ne* (5) and without *ne* (6).

*(5)*  *Raam= **ne** samaachaar bolaa*
    Ram=erg news tell-Msc.Sg.Prf
    Ram told the news.
*(6)*  *Raam samaachaar bolaa*
    
*Ram news tell-Msc.Sg.Prf*
    Ram told the news.

Although previous naturalistic and experimental studies of Hindi children’s ergative marking have been conducted (e.g., Narasimhan, 2005; Pareek *et al*., 2016) both focussed on errors of omission, rather than the question of how children learn *exceptions* to general patterns of ergative marking; the key question from a learnability perspective.

How, then, do children learn this partial regularity? The previous studies discussed above (mainly focussing on English) have provided evidence for two complementary possibilities. Under the first – statistical preemption (e.g., Goldberg, 1995; Goldberg, 2006; Goldberg, 2011; Goldberg, 2019) – children are sensitive to the probabilistic competition between forms with the same meaning. For example, the overgeneralized forms *foots* and *mans* are outcompeted by repeatedly hearing *feet* and *men*, while the overgeneralized form *[A] danced [B]* (e.g., *Somebody danced the boy) is outcompeted by repeatedly hearing *[A] made [B]* dance (e.g., *Somebody made the boy dance*). When applied to Hindi ergative marking, preemption holds that via repeatedly hearing (active, past tense/perfective aspect, transitive) sentences containing a particular verb but without *ne* marking (example 3), these “zero-marked” subjects come to outcompete *ne* marked subjects for that verb (example 4). Thus the predictions that we test in the present study are that the greater the frequency with which a particular verb appears with versus without *ne* marking on the subject – relative to other verbs – the greater the extent to which (a) - *ne* marked forms will be preferred over zero-marked forms in a judgment task (Study 1) and (b) *-n*e marked forms will be produced over zero-marked forms in an elicited production task (Study 2).

 The second possibility – again supported by previous studies of English and a handful of other languages (e.g., Ambridge *et al*., 2020; Ambridge *et al*., 2022; Gropen *et al*., 1989; Gropen *et al*., 1991) – is that children might be guided by semantics. For example, at least part of the reason that English speakers prefer *Somebody made the boy dance* over * *Somebody danced the boy* is that “dancing” is not really an action that is amenable to outside causation: someone can *ask* a boy to dance, but not force him, he retains some agency. In contrast, English speakers accept both *Somebody made the water boil* and *Somebody boiled the water* because this type of direct, outside causation is possible ( Ambridge *et al*., 2020; Ambridge *et al*., 2022; Pinker, 1989; Shibatani & Pardeshi, 2002). In the domain of Hindi ergative *ne* marking, effects of semantics might be observed at either or both of two levels: the verb (lexical) and the clause (clausal). At the lexical level, linguistic analyses of Hindi suggest that the higher a verb on a cline of semantic transitivity based on the type of action it denotes, the greater the likelihood that it triggers ergative *ne* marking on the subjects (e.g., *break, squash, eat* > *fear, escape, find*; Hopper & Thompson, 1980; Mohanan, 1994; Montaut, 2013). Intentionality (or volitionality) at the clausal level can also contribute to higher transitivity ( Hopper & Thompson, 1980): events can be presented as intentional versus unintentional by the use of adverbs that modify the action denoted by the verb (7).

*(7)*  *Mohan= **ne** rassii-0 khiinc-ii galtii se.*
    Mohan=Erg rope-Nom pull-Fem.Sg.Prf mistake by.
    ‘Mohan pulled the rope by mistake’

On the other hand, verbs that are lower on the transitivity cline mark subject arguments with non-canonical case-marking ( Montaut, 2013). For example, two-participant verbs that denote states (versus actions) typically have subject arguments that are low in agency (e.g., experiencers, recipients, possessors) and object arguments that are not highly affected. In such cases, subjects receive cases such as dative, locative, or genitive ( Mohanan, 1994; Montaut, 2013).

*(8)*  *Mohan=ko kitaab-0 caahiye*
    Mohan=Dat book-Nom want/need
    ‘Mohan wants/needs a book.
*(9)*  *niinaa=mE apnii mausii=keliye baRii mamtaa-0 hae.*
    Nina=Loc self aunt=for much affection-Nom be.3.Sg.Pres
    ‘Nina has a lot of affection for her aunt.’ ( Mohanan, 1994:181)

In terms of the retreat from overgeneralization, then, the verb-level semantics hypothesis holds that children set up a semantically-restricted generalization: that only verbs that are high on this cline of transitivity trigger ergative *ne*- marking (in otherwise suitable contexts).

In the present study, we operationalize this notion of transitivity in verb-level semantics in terms of the degree to which the patient is affected by the action denoted by the verb ( Hopper & Thompson, 1980). We ask adult participants to rate sentences describing various actions for “the extent to which the [THING] gets affected or changed by the event in some way”. The prediction of a verb-level semantics account is that the higher the rated transitivity, the greater the extent to which *ne* versus zero-marked subjects of the verb, as depicted by the corresponding action in the videos, will be judged as acceptable (Study 1) and produced (Study 2).

However, it is far from clear that a verb-level semantics effect exists for Hindi. Indeed, as a reviewer, Aaricia Ponnet, pointed out, by restricting the present study to two-participant transitive verbs, we have already simplified the task facing learners: Although ergative case-marking is *predominantly* found with two-participant verbs, it can also optionally occur on the single argument of a small set of intransitive verbs (e.g., *chiikh* ‘scream’), apparently marking a volitional action (e.g., Butt & King, 1991; Butt & King, 2003; de Hoop & Narasimhan, 2005; Mohanan, 1994). And, of course, as Ponnet highlights, learning this “morphosyntactic opposition between ergative and nominative” – as it applies across both intransitive and transitive verbs – is “elementary…to the learnability of split ergativity as a whole”. Future studies, then, should include this full range of verbs.

Focussing on the present set of transitive verbs, these considerations raise the possibility that semantic effects on Hindi ergative *ne* marking, if they exist at all, may occur not at the verb level, but at the clausal level: whether the action denoted by a verb is presented as intentional or unintentional. In terms of the retreat from overgeneralization, then, the clausal-level semantics hypothesis holds that children set up a semantically-restricted generalization: that only actions that are clearly intentional trigger ergative *ne*-marking (in otherwise suitable contexts). In terms of the present study then, the prediction is that presenting an action as intentional rather than unintentional will increase the extent to which *ne* marked forms are – relative to zero-marked forms –rated as acceptable. Note that since none of the theoretical proposals tested make any claims regarding effects of participant sex and/or gender, this information was not recorded.

## Study 1: Grammatical acceptability judgments

The sample size, methods and data-analysis plan were registered on the Open Science Framework prior to data collection ( Rosenthal, 1979). 

### Participants

For the grammaticality judgment task, 48 participants from each of three age groups, 5;6–6;6 ( *M*: 5.90, *SD*: 0.30), 9;6–10;6 ( *M*: 10.06, *SD*: 0.33) ^2^ and adults ( *M*: 22.01, *SD*: 2.60), took part in the study. A further 20 adult speakers completed the semantic ratings task ( *M*: 29.81, *SD*: 9.15). These sample sizes were chosen based on similar research conducted previously ( Ambridge *et al*., 2020) and because of time and financial constraints (as specified on the relevant grant application). For recruitment, eight schools and two universities were approached in Jabalpur, Bhopal and Delhi in India. Five schools and both the universities agreed to take part in the main study. In total, including online data – see below – data was collected from 100 children aged 9;6–10;6 and 5;6–6;6 (9;6–10;6: 48 data points used, 2 excluded (incorrect lists was administered for one and one child withdrew; 5:6–6;6: 48 data points used, 2 excluded (due to collecting more than our preregistered total)) and 73 adults (Grammatical judgment task: 48 data points used, 4 excluded (4 data points were not saved due to technical issues; Semantics ratings study: 20 data points used, 1 excluded (participant withdrew)).

COVID-19 pandemic restrictions came into force ( GOV.UK, 2022; UK Parliament, 2021) before face-to-face testing could be completed and therefore, some participants were recruited via the online recruitment portals Prolific and Amazon Mechanical Turk: For the judgment task, data for all 48 adults and 45/48 children aged 9;6–10;6 were collected in face-to-face sessions. Data from 3/48 children aged 9;6–10;6 and 48 /48 children aged 5;6–6;6 were collected in online sessions. All the data for the adult semantics ratings task (20/20 participants) were collected in online sessions. As part of a pilot study to refine the tasks, ten adults completed the grammaticality judgment task and two adults completed the semantics rating task (all face-to-face). These data are not included in the main analyses.

Children and adults with Hindi as their first language and no known history of speech and language impairments were eligible to take part in the study. All participants had Hindi as their first language and knew one or more additional language. No monetary incentives were provided to schools, though children received stickers and pencils as rewards when data were collected in face-to-face sessions. Parents/caregivers who assisted their children during online data collection received £5 (Indian Rupee equivalent) as compensation for their time and effort. For the grammaticality judgment and the semantic ratings tasks, adult participants received 200 INR and £7.50 GBP compensation respectively.

### Materials

***Verbs.*** Forty action verbs were chosen from the Action/Process category of Concepticon ( List *et al*., 2016), a database of concepts that are commonly lexicalized as words across languages (e.g., *run, jump, dance*) and two published sources on Hindi: Piepers (2016) and Mohanan (1994). Eligible verbs had to be (a) transitive verbs that took an ergative or nominative subject (b) familiar to young children, and (c) easily depictable in animations. In total, according to the intuitions of two native speakers of Hindi, of the 40 verbs, 25 verbs occur with the ergative *ne* marker on the subject, 5 verbs occur with zero-marking and 10 verbs exhibited optional *ne* marking (versus zero-marking). All verbs and sentences are listed in the Appendix.

***Sentences.*** For each verb, four sentence structures were generated which were manipulated for the (a) presence or absence of the ergative *ne* marker on the subject (7a & 7b), and (b) indicating intentionality of the agent performing the action (7a and 7b show the more intentional situations, 7c & 7d the less intentional situations by use of an adverbial phrase *galtii se*). All sentences were in the form of [AGENT] [PATIENT] [VERB], where the agent was always *The boy* and the patient an inanimate ^3^ masculine noun (to ensure uniform verb agreement marking: masculine, singular perfective).

7a. *laRke=ne khel-0 jiit-aa*
    boy=Erg game-Nom win-Msc.Sg.Prf
    The boy won the game
7b. *laRkaa-0 khel-0 jiit-aa*
    The boy-Nom game-0 win-Msc.Sg.Prf
    The boy won the game
7c. *laRke=ne galtii=se khel=0 jiit-aa*
    The boy=Erg mistake=Inst game-Nom win-Msc.Sg.Prf
    The boy won the game by mistake
7d. *laRkaa-0 galtii=se khel-0 jiit-aa*
    The boy-Nom mistake=Inst game-Nom win-Msc.Sg.Prf
    The boy won the game by mistake

***Animations.*** The animations were created using Moho Debut 12. This is proprietary software, and no free alternative is available. However, a free trial version is available, and the animations themselves are encoded as mp4 files, which can be viewed using a wide range of free software packages.

For each verb, two different animations were created, one depicting the intentional and the other the unintentional event (all performed by the same boy character). For the animations, see *Underlying data* ( Ambridge *et al*., 2023a).

For the grammaticality judgment task (Study 1), the animations were accompanied by the relevant sentence structure as described above. The sentences were recorded by a male Hindi speaker using the freeware package Audacity 2.1.2 ( Audacity Team, 2016). Seven different animations were used in the practice trials of the grammaticality judgment task ( Ambridge *et al*., 2023a).

### Procedure

***Grammaticality judgment task.*** The face-to-face grammaticality judgment task was administered in line with the procedure outlined in Ambridge *et al*. (2008: 105–107), using the free Open Source platform PsychoPy2 ( Peirce *et al.*, 2019). In brief, the participant played a game with a talking dog (who produces the sentences via a loudspeaker) and were asked to assist the dog in “learning Hindi”. On each trial the participant and the talking dog watched a video together and the dog provided a description of the action. The participant gave feedback to the dog on the acceptability of the sentence produced (grammatical/ungrammatical) by selecting either a red counter (to indicate ungrammatical) or a green counter (to indicate grammatical), then placing this counter on a five-point smiley face scale ranging from sad (red) to happy (green) to indicate the degree of (un)grammaticality. Due to the COVID-19 pandemic, part of the data (48 children aged 5;6–6;6 & 3 children aged 9;6–10;6) was collected online using Gorilla, a platform where experiments can be built for free and fees are paid for data collection. There is no free alternative. For the online version, parents/caregivers were asked to assist the child in understanding the task, completing the practice trials, and inputting children’s answers. However, they were asked to refrain from prompting or assisting their children when completing the main trials.

For adults ( *N*=48), the design was completely within subjects. For each of 40 verbs, adults rated the grammatical acceptability of four sentences (and the accompanying videos), crossing ergative subject marking (with *ne*/without *ne*) and intentionality (intentional/unintentional), for a total of 160 trials in one session which took approximately 30 minutes to complete (see examples 7a–d).

As children would have found it difficult to complete 160 trials, a reduced set of 20 verbs (5 obligatory *ne* verbs, 5 obligatory zero marked verbs, and 10 optional verbs, according to the intuition of two native-speakers) was chosen from the larger set of 40. Since even 80 trials (20 verbs x 4 sentence types) would have been too many for young children, two counterbalance lists - each containing 15 verbs were created. The 10 verbs for which *ne* marking is somewhat “optional” ( *know, leap-over, lose, find, talk nonsense, sing, smell, speak, understand* and *win*) were included in both lists because they are the verbs that are most likely to show sensitivity to our preemption and semantics predictors, as well as the intentionality manipulation. The 10 verbs which occur with *ne* or zero marking (according to native speaker intuitions) were considered to be less sensitive to the experimental manipulations, so were split across the two lists. i.e., List 1 comprised 2 obligatory verbs and 3 zero verbs (as well as the 10 optional verbs); list 2 comprised 3 obligatory verbs and 2 zero verbs (as well as the 10 optional verbs). Thus, each child provided ratings for all four sentence structures for 15 verbs (60 trials in total). For the full verb lists, see Underlying data ( Ambridge *et al*., 2023a). Children completed the trials over two sessions which took place either on the same day after a break or on two separate days. 

Trials were presented in a random order. Prior to the main task, all participants completed a training session during which they received feedback for seven sentences with varying degrees of acceptability (which were Hindi translations of the sentences used in a study with English-acquiring children in Ambridge *et al*., 2008).

***Semantic ratings task.*** As mentioned in the pre-registration, for the semantic ratings task, participants viewed all 80 animations (the same animations as in the judgment task; both intentional and unintentional) along with the corresponding verb and the patient argument that could be used to describe the action and provided ratings for patient affectedness. However, the results that we obtained did not seem, based on our native intuitions, to really capture the relevant notion of affectedness and hence a revised, simplified version of the task was used instead. The modified semantic ratings task ( *N*=20, adults only) was also completely within-subjects. Participants were given 40 sentences in the passive form describing an action (e.g. “ *The potato was cooked”*) and rated the extent to which the object was affected or changed in the action, according to the following instructions (translated into Hindi). They did not view animations along with the sentences.

*Thanks for taking part in this experiment.*

*In total, you will see 40 sentences.*

*Each sentence will describe an action that has been carried out.*

*Please rate the extent to which the [THING] gets affected or changed by the event in some way.*

Participants were asked to provide their ratings using a visual analogue scale which ranged from

*Not at all-----------------------------------------------------------Very much so*

Prior to the main trials, participants completed three practice trials. The materials for the main and practice trials can be accessed on the OSF project link in the folder titled ‘Ergative_SemanticsTaskMaterials’.

It is important to acknowledge and highlight the fact that, as pointed out by a reviewer, Aaricia Ponnet, this semantic rating task measures just *one* aspect of potentially-relevant semantics: patient affectedness. Theoretical analyses of transitivity highlight its multifactorial nature, with transitivity affected not only by the “degree of affectedness of the patient”, but also “the effectiveness with which an action takes place, the punctuality and telicity of the verb, the conscious activity of the agent, [and] the referentiality…of the object” (e.g., Hopper & Thompson, 1980: 251). Although our additional intentionality manipulation investigates the effect of “conscious activity of the agent” (as we note in the Discussion, only to a limited extent), future studies should address the other semantic factors that are likely to be relevant to learning ergative *-ne* versus zero marking.

### Predictors

***Preemption.*** The prediction that follows from preemption is that the greater the frequency with which a particular verb appears with versus without *ne* marking on the subject – relative to other verbs in the test set – the greater the extent to which *ne* marked forms will be preferred over zero-marked forms in the judgment task. As in previous studies ( Ambridge *et al*., 2018; Ambridge *et al*., 2020) we operationalized this measure using a scaled and centred chi-square statistic which measures how often, in past tense/perfective aspect active sentences, a particular verb triggers ergative case marking as compared with all other verbs in the test set of 40, e.g., (example figures only):

**Table:** 

	Subject with *ne*	Subject without *ne*
**Target verb** **All other verbs**	2 10,000	10 1,500

The frequency counts were obtained from the Hindi monolingual corpus, Indic NLP Suite: Monolingual Corpora ( Kakwani *et al*., 2020) which consists of 1 million sentences. The corpus was parsed using the Hindi parser ( Bhat *et al*., 2017) and the counts for subjects marked with the *ne* ergative and zero markers were extracted. The chi-square method for calculating the preemption predictor was exactly the same as in Ambridge *et al*. (2018).

A reviewer, Vsevolod Kapatsinski, suggested that this chi-square measure “conflates frequency in the rated construction (e.g., with *ne*) and frequency in the other construction (without *ne*). The latter is an implementation of preemption but the former is not. How do we know that frequency in the other construction matters for the judgments?”. We accept that because preemption was originally presented as a descriptive verbal theory (i.e., not an equation or computational model) there is room for disagreement about precisely how it should be instantiated numerically. However, we maintain that any valid measure of preemption must reflect the *relative* corpus frequency of the form that occurred (here, a *ne*-marked noun) and a form that feasibly *could have but did not* occur (here, a zero-marked noun). This is clear in, for example, the following definition from Goldberg (2011), with Hindi examples added in square brackets:

In a situation in which a construction A (CxA) might have been expected to be uttered with a given verb, *verb*_i_, the learner can infer that CxA [e.g., *laRke=ne*, ‘boy-Erg’] is not after all appropriate for that verb, if a different construction, CxB [e.g., *laRkaa-0*, ‘boy-Nom’], is consistently witnessed instead.

Indeed, a version of preemption (or learning/reasoning more generally) that did not take into account the “base rate” in this way would be very difficult to support on logical grounds; like arguing that Drug A (5 deaths amongst 50 patients) is safer than Drug B (10 deaths amongst 1,000 patients). For this reason, we follow previous studies that have used the chi-square/Fisher’s exact method for calculating the measure of preemption/competition (e.g., Ambridge *et al*., 2018; Ambridge *et al*., 2020; Ambridge *et al*., 2022; Ambridge *et al.*, 2023; Liu & Ambridge, 2021; Stefanowitsch, 2008; Tatsumi *et al*., 2018a; Tatsumi *et al*., 2018b).

***Semantics.*** Verb and clause-level semantics were measured using patient affectedness ratings and the intentionality manipulation respectively. To create the patient affectedness predictor for each verb, we took the mean rating (on the 100-point visual-analogue scale) on the semantics ratings task described above across all 20 participants, then scaled and centred the mean ratings. The binary intentionality manipulation was instantiated by having participants rate intentional and unintentional versions of the sentences (see examples 7a–d) on the judgment task. We did not investigate the effect of intentionality on the *production* of *ne* marking (the subsequent Study 2) since children might not be able to infer the intentionality of an action from the animations alone (recall that in the judgment task, the relevant sentences included the term “unintentionally”, see examples 7a–d). It is important to also emphasize that the intentional and unintentional versions of each sentence were paired with different videos. For example, the intentional version of *The boy soaked the cloth* was illustrated by the boy dipping the cloth into water, while the unintentional version was illustrated by a girl knocking the boy, forcing him to unintentionally drop the cloth into water.

### Qualitative analyses

**Figure 1.**
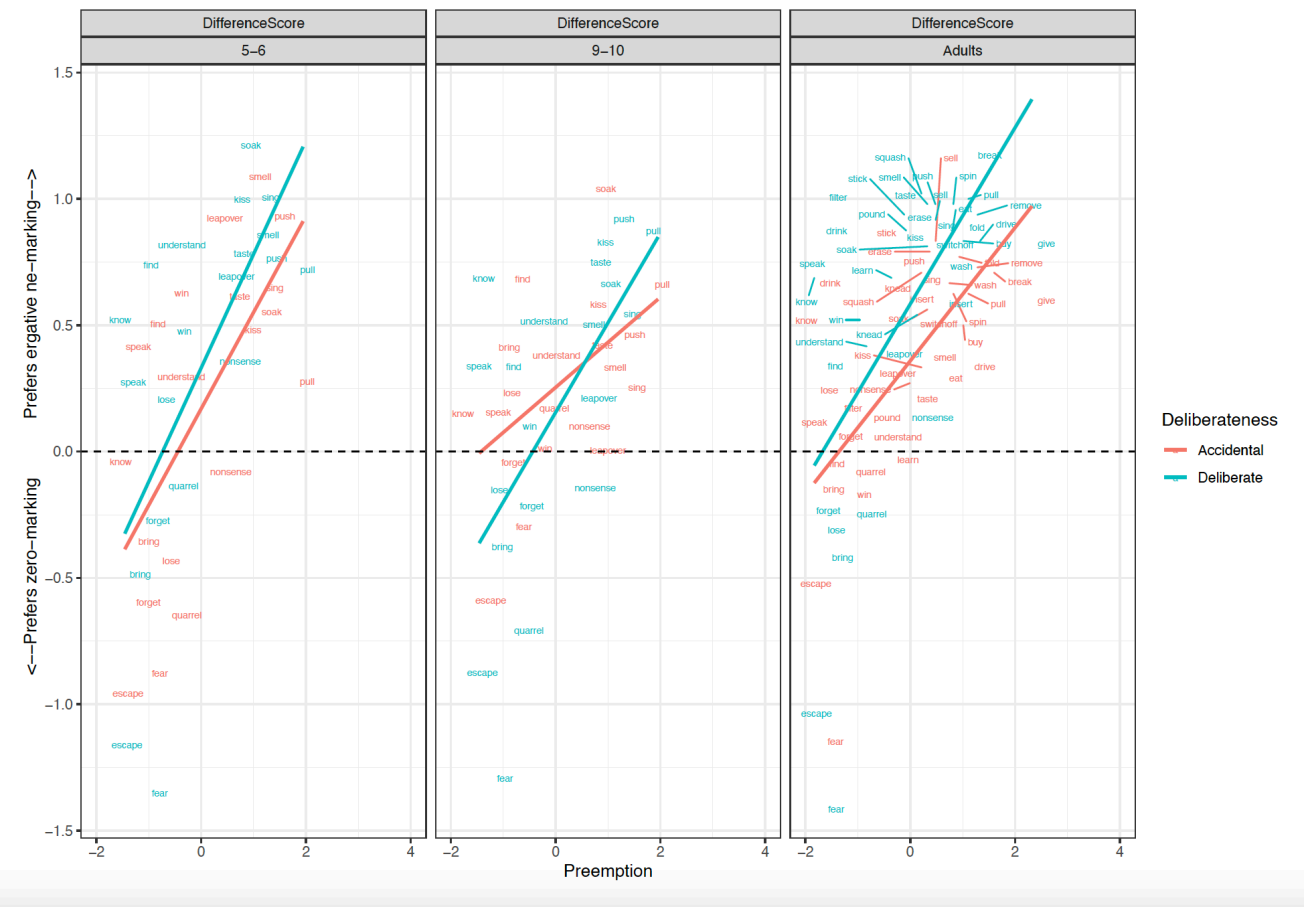
Relationship between Preemption counts and Difference scores across clause-level-semantics conditions and age groups. Figure 1 shows the relationship between preemption counts (higher corpus relative verb frequency triggering ne form) and difference scores across clause-level-semantics conditions (Intentional=Deliberate, Unintentional=Accidental) and age groups (5;6–6;6. 9;6–10;6, Adults).

Before turning to the preregistered statistical analyses, it is informative to first briefly discuss the qualitative patterns observed (see Figure 1– Figure 4), particularly with regard to the verb- and clause-level semantic manipulations, seen most clearly in Figure 3 (for discussion and analysis of Hindi children’s acquisition of ergative case marking “in the wild” see Narasimhan, 2005). Looking first at the adult data (rightmost panel of Figure 3), one of the most striking findings is that while only a handful of verbs prefer zero marking, we see a smooth cline from verbs that prefer *-ne* marking very strongly (e.g., *break, filter, smell, spin, push*) to those that prefer *-ne* over zero marking only marginally, particularly when the action is non-intentional (e.g., *speak, understand, lose, talk nonsense*). Also intriguing is the pattern shown by verbs such as *win* and *lose*, which prefer *ne-* marking for intentional actions, but flip to a preference for zero marking for unintentional actions. This suggests that ergative marking is multifactorial and probabilistic, and cannot be captured by a straightforward division of verbs into those that trigger ne-/zero/optional marking. The child data are similar to the adult data in that both child groups seem to show broadly-adultlike effects of both patient-affectedness and intentionality (particularly for zero-preferring verbs such *as fear, escape, quarrel* and *forget*), though with a few exceptions. For example, both child groups overestimate – relative to adults – *-ne* preference for soak; the older children particularly so for unintentional soaking.

### Statistical analyses

The data were analysed using R version 3.6.3 (R Studio Version 1.2.5042) ( R Core Team, 2020). Mixed effects models were run using the lme4 package ( Bates *et al*., 2015); *p* values were obtained using lmerTest ( Kuznetsova *et al.*, 2017). Statistical analyses were conducted for each age group separately (5;6–6;6; 9;6–10;6; Adults). We did not compare directly across groups since the tasks are not strictly comparable for the adults and children who completed different verb sets.

All analyses were conducted according to our pre-registered analysis plan ^4^. Two aspects of this plan are particularly important to highlight. First, the main set of analysis uses *difference scores*: preference for the *ne* over zero marked form of the subject argument of each verb, within each intentionality pair, within each participant. These scores control for general (dis-)preferences that participants may show for particular verbs and/or videos. However, for the sake of completeness, we also report analyses conducted on the raw *ne* and zero-marked forms. Second, we report both (a) simultaneous models, in which the preemption, verb-level semantics (patient affectedness) and clause-level semantics (intentionality) are all included and (b) single-predictor models, each of which includes only preemption OR verb-level semantics (along with intentionality). This is necessary because we expected a high degree of collinearity between these predictors: Indeed, a by-verb correlation analysis revealed moderate correlations between the verb-semantics and preemption predictors: *r*=.40 for the child set, and *r*=.26 for the larger adult set.

### Results

Table 1– Table 9 show, for each age-group, the simultaneous and single-predictor models for ( Table 1– Table 3) Difference scores, ( Table 4– Table 6) raw *ne* marked forms and ( Table 7– Table 9) raw zero-marked forms. The ratings are plotted in Figure 1– Figure 4.

**Table 1.** Difference Scores: Simultaneous and Single-predictor Models for 5–6 year olds.

	*Est*	*SE*	*df*	*t*	*p*
**Simultaneous ^a^**					
Intercept	0.19	0.12	25.07	1.51	0.14
** Preemption**	**0.43**	**0.13**	**22.02**	**3.30**	**0.00**
Semantics	-0.08	0.12	22.07	-0.68	0.50
** Intentionality**	**0.17**	**0.08**	**1369.10**	**2.13**	**0.03**
Semantics*Intentionality	0.06	0.09	1369.10	0.73	0.46
Intentionality*Preemption	-0.01	0.09	1369.10	-0.08	0.94
**Single-predictor (Preemption)**					
Intercept	0.18	0.12	21.67	1.52	0.14
** Preemption**	**0.39**	**0.11**	**20.00**	**3.42**	**0.00**
Intentionality	0.17	0.08	17.33	2.05	0.06
Preemption*Intentionality	0.02	0.08	18.94	0.28	0.79
**Single-predictor (Semantics)**					
Intercept	0.18	0.15	19.77	1.16	0.26
Semantics	0.11	0.14	22.38	0.76	0.46
Intentionality	0.17	0.08	17.80	2.04	0.06
Semantics*Intentionality	0.07	0.09	26.67	0.71	0.48

^a^The fully maximal simultaneous model failed to converge and so, the model was run excluding random effects of intentionality.

**Table 2.** Difference Scores: Simultaneous and Single-predictor Models for 9–10 year olds.

	*Est*	*SE*	*df*	*t*	*p*
**Simultaneous**					
Intercept	0.24	0.09	30.95	2.79	0.01
Preemption	0.13	0.08	21.24	1.59	0.13
Semantics	0.07	0.08	25.16	0.85	0.40
Intentionality	-0.07	0.10	25.06	-0.75	0.46
Semantics*Intentionality	-0.07	0.10	28.62	-0.67	0.51
Intentionality*Preemption	0.20	0.11	22.61	1.89	0.07
**Single-predictor ^a^ (Preemption)**					
Intercept	0.24	0.10	41.06	2.31	0.03
Preemption	0.18	0.09	28.00	2.04	0.05
Intentionality	-0.05	0.08	1371.13	-0.70	0.49
Preemption*Intentionality	0.13	0.08	1371.13	1.68	0.09
**Single-predictor ^a^ (Semantics)**					
Intercept	0.23	0.11	33.77	2.04	0.05
Semantics	0.15	0.09	25.29	1.58	0.13
Intentionality	-0.05	0.08	1370.11	-0.69	0.49
Semantics*Intentionality	-0.02	0.08	1370.11	-0.25	0.80

^a^The fully maximal single-predictor models failed to converge and so, the model was run excluding random effects of intentionality.

**Table 3.** Difference Scores: Simultaneous and Single-predictor Models for Adults.

	*Est*	*SE*	*df*	*t*	*p*
**Simultaneous**					
Intercept	0.36	0.08	62.84	4.64	0.00
** Preemption**	**0.24**	**0.06**	**53.32**	**4.09**	**0.00**
Semantics	0.08	0.05	43.27	1.53	0.13
** Intentionality**	**0.23**	**0.07**	**45.41**	**3.42**	**0.00**
Semantics*Intentionality	0.01	0.06	36.77	0.15	0.88
Intentionality*Preemption	0.08	0.06	38.45	1.48	0.15
**Single-predictor (Preemption)**					
Intercept	0.36	0.08	63.86	4.60	0.00
** Preemption**	**0.26**	**0.06**	**56.18**	**4.36**	**0.00**
** Intentionality**	**0.23**	**0.06**	**44.82**	**3.47**	**0.00**
Preemption*Intentionality	0.09	0.05	39.29	1.58	0.12
**Single-predictor (Semantics)**					
Intercept	0.36	0.09	68.64	4.19	0.00
** Semantics**	**0.14**	**0.07**	**48.15**	**2.17**	**0.03**
** Intentionality**	**0.23**	**0.07**	**44.05**	**3.43**	**0.00**
Semantics*Intentionality	0.03	0.06	39.24	0.53	0.60

**Table 4.** Raw Ne Marked Forms: Simultaneous and Single-predictor Models for 5–6 year olds.

	*Est*	*SE*	*df*	*t*	*p*
**Simultaneous**					
Intercept	3.50	0.14	33.99	24.81	0.00
** Preemption**	**0.33**	**0.13**	**18.76**	**2.54**	**0.02**
Semantics	-0.05	0.13	20.38	-0.43	0.67
** Intentionality**	**0.66**	**0.11**	**30.57**	**5.96**	**0.00**
Semantics*Intentionality	0.06	0.10	21.95	0.56	0.58
Intentionality*Preemption	-0.06	0.10	17.86	-0.59	0.56
**Single-predictor (Preemption)**					
Intercept	3.50	0.14	36.54	25.30	0.00
** Preemption**	**0.30**	**0.11**	**20.29**	**2.70**	**0.01**
** Intentionality**	**0.66**	**0.11**	**31.59**	**6.10**	**0.00**
Preemption*Intentionality	-0.03	0.09	20.10	-0.39	0.70
**Single-predictor (Semantics)**					
Intercept	3.50	0.15	31.46	22.69	0.00
Semantics	0.09	0.12	20.99	0.75	0.46
** Intentionality**	**0.66**	**0.11**	**31.42**	**6.08**	**0.00**
Semantics*Intentionality	0.03	0.09	25.93	0.34	0.74

**Table 5.** Raw Ne Marked Forms: Simultaneous and Single-predictor Models for 9–10 year olds.

	*Est*	*SE*	*df*	*t*	*p*
**Simultaneous**					
Intercept	3.69	0.12	45.15	31.58	0.00
Preemption	0.15	0.09	18.42	1.62	0.12
Semantics	0.03	0.09	19.10	0.36	0.73
** Intentionality**	**0.57**	**0.09**	**34.91**	**6.35**	**0.00**
Semantics*Intentionality	-0.11	0.08	27.05	-1.36	0.19
Intentionality*Preemption	0.12	0.08	25.44	1.40	0.17
**Single-predictor (Preemption)**					
Intercept	3.69	0.11	48.20	32.29	0.00
Preemption	0.16	0.08	20.57	1.98	0.06
** Intentionality**	**0.57**	**0.09**	**35.77**	**6.26**	**0.00**
Preemption*Intentionality	0.08	0.08	27.57	1.00	0.33
**Single-predictor (Semantics)**					
Intercept	3.69	0.12	44.43	30.88	0.00
Semantics	0.10	0.08	20.92	1.19	0.25
** Intentionality**	**0.57**	**0.09**	**31.87**	**6.23**	**0.00**
Semantics*Intentionality	-0.05	0.07	27.30	-0.69	0.50

**Table 6.** Raw Ne Marked Forms: Simultaneous and Single-predictor Models for Adults.

	*Est*	*SE*	*df*	*t*	*p*
**Simultaneous**					
Intercept	3.84	0.10	75.11	37.59	0.00
** Preemption**	**0.29**	**0.07**	**44.01**	**4.15**	**0.00**
Semantics	0.06	0.07	43.68	0.88	0.38
** Intentionality**	**0.65**	**0.10**	**70.12**	**6.39**	**0.00**
Semantics*Intentionality	0.00	0.07	39.88	0.06	0.95
Intentionality*Preemption	-0.06	0.07	41.16	-0.89	0.38
**Single-predictor (Preemption)**					
Intercept	3.84	0.10	75.56	37.62	0.00
** Preemption**	**0.30**	**0.07**	**47.66**	**4.46**	**0.00**
** Intentionality**	**0.65**	**0.10**	**70.36**	**6.43**	**0.00**
Preemption*Intentionality	-0.06	0.07	42.56	-0.91	0.36
**Single-predictor (Semantics)**					
Intercept	3.84	0.11	78.66	34.57	0.00
Semantics	0.13	0.08	44.84	1.66	0.10
** Intentionality**	**0.65**	**0.10**	**70.52**	**6.41**	**0.00**
Semantics*Intentionality	-0.01	0.07	41.60	-0.18	0.86

**Table 7.** Raw Zero Marked Forms: Simultaneous and Single-predictor Models for 5–6 year olds.

	*Est*	*SE*	*df*	*t*	*p*
**Simultaneous**					
Intercept	3.31	0.11	39.21	29.68	0.00
Preemption	-0.10	0.10	18.79	-1.03	0.31
Semantics	0.04	0.09	21.40	0.41	0.69
** Intentionality**	**0.52**	**0.12**	**26.37**	**4.25**	**0.00**
Semantics*Intentionality	-0.02	0.12	20.43	-0.21	0.84
Intentionality*Preemption	-0.07	0.12	17.43	-0.63	0.53
**Single-predictor (Preemption) ^a^**					
Intercept	3.32	0.11	47.71	30.08	0.00
Preemption	-0.08	0.09	26.12	-0.95	0.35
** Intentionality**	**0.50**	**0.06**	**1327.96**	**8.05**	**0.00**
Preemption*Intentionality	-0.08	0.06	1327.96	-1.26	0.21
**Single-predictor (Semantics)**					
Intercept	3.31	0.11	40.51	29.68	0.00
Semantics	-0.01	0.08	23.85	-0.07	0.94
** Intentionality**	**0.52**	**0.12**	**27.64**	**4.32**	**0.00**
Semantics*Intentionality	-0.06	0.10	23.00	-0.57	0.58

^a^The fully maximal single-predictor model failed to converge and so, the model was run excluding random effects of intentionality.

**Table 8.** Raw Zero Marked Forms: Simultaneous and Single-predictor Models for 9–10 year olds.

	Est	SE	df	t	p
**Simultaneous**					
Intercept	3.45	0.11	46.68	30.90	0.00
Preemption	0.01	0.08	20.69	0.09	0.93
Semantics	-0.04	0.08	19.36	-0.54	0.60
** Intentionality**	**0.64**	**0.09**	**26.92**	**7.16**	**0.00**
Semantics*Intentionality	-0.05	0.07	14.95	-0.64	0.53
Intentionality*Preemption	-0.06	0.08	14.32	-0.84	0.42
**Single-predictor (Preemption)**					
Intercept	3.45	0.11	48.45	31.30	0.00
Preemption	-0.02	0.07	24.08	-0.20	0.84
** Intentionality**	**0.64**	**0.09**	**27.61**	**7.25**	**0.00**
Preemption*Intentionality	-0.08	0.07	17.14	-1.21	0.24
**Single-predictor (Semantics)**					
Intercept	3.45	0.11	47.96	31.31	0.00
Semantics	-0.04	0.07	22.25	-0.59	0.56
** Intentionality**	**0.64**	**0.09**	**26.95**	**7.30**	**0.00**
Semantics*Intentionality	-0.07	0.06	17.43	-1.16	0.26

**Table 9.** Raw Zero Marked Forms: Simultaneous and Single-predictor Models for Adults.

	*Est*	*SE*	*df*	*t*	*p*
**Simultaneous**					
Intercept	3.48	0.09	69.23	40.84	0.00
Preemption	0.04	0.06	49.10	0.77	0.45
Semantics	-0.02	0.05	39.00	-0.43	0.67
** Intentionality**	**0.42**	**0.09**	**61.03**	**4.85**	**0.00**
Semantics*Intentionality	-0.00	0.05	38.43	-0.08	0.94
Intentionality*Preemption	**-0.15**	**0.05**	**40.41**	**-2.70**	**0.01**
**Single-predictor** **(Preemption)**					
Intercept	3.48	0.08	69.20	40.99	0.00
Preemption	0.04	0.06	51.69	0.69	0.49
** Intentionality**	**0.42**	**0.09**	**60.77**	**4.88**	**0.00**
Preemption*Intentionality	**-0.15**	**0.05**	**42.48**	**-2.80**	**0.01**
**Single-predictor (Semantics)**					
Intercept	3.48	0.09	69.27	40.92	0.00
Semantics	-0.01	0.05	40.02	-0.22	0.83
** Intentionality**	**0.42**	**0.09**	**63.98**	**4.72**	**0.00**
Semantics*Intentionality	-0.04	0.06	39.52	-0.75	0.46

**Figure 2.**
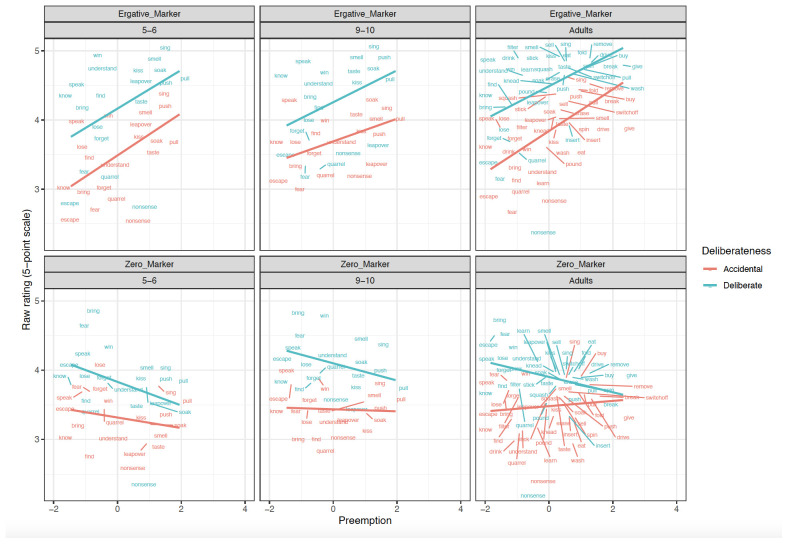
Relationship between Preemption counts and Raw scores across clause-level-semantics conditions and age groups. Figure 2 shows the relationship between preemption (higher corpus relative verb frequency triggering ne form) counts and raw scores across clause-level-semantics conditions (Intentional=Deliberate, Unintentional=Accidental) and age groups (5;6–6;6. 9;6–10;6, Adults).

**Figure 3.**
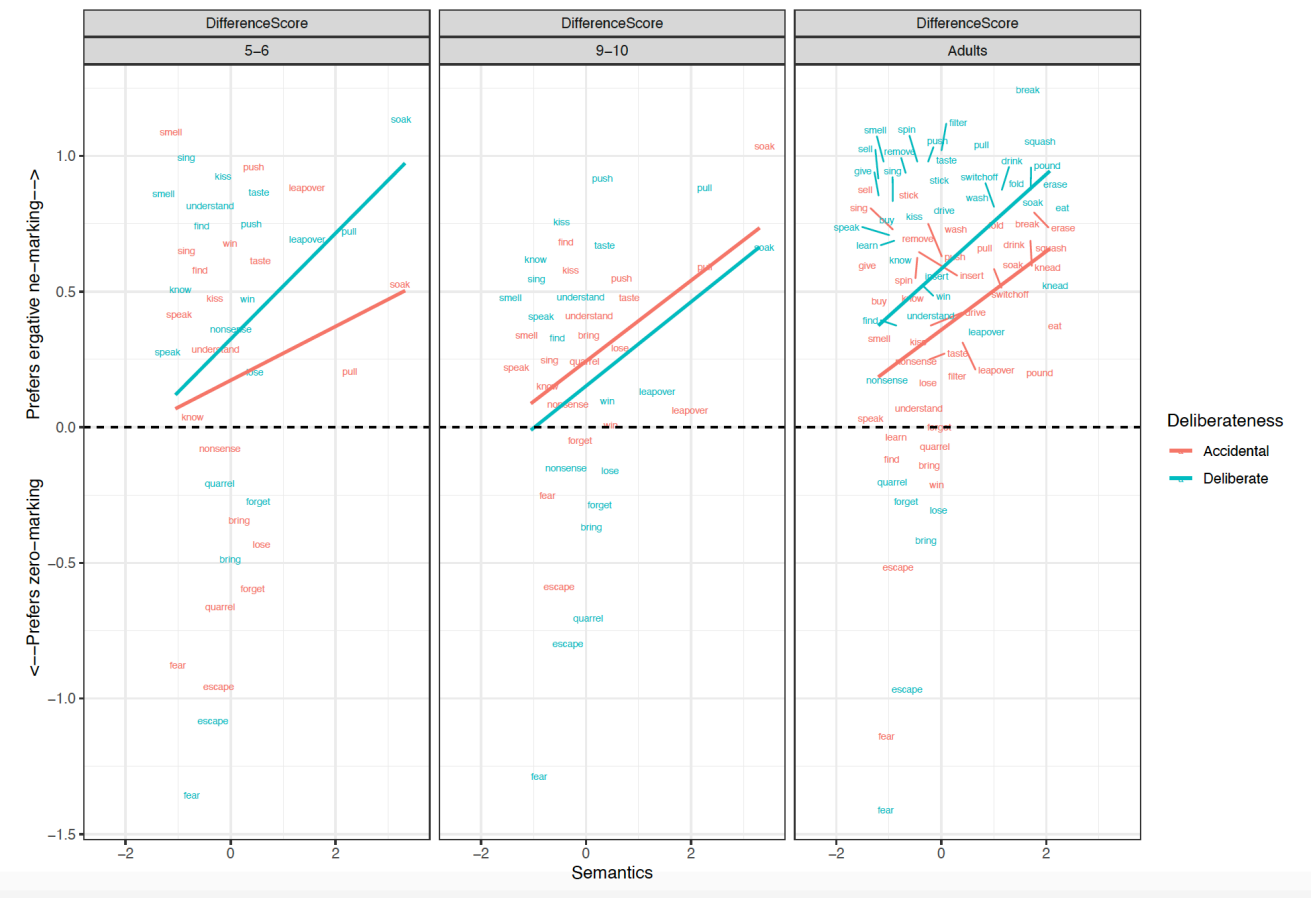
Relationship between verb-level Semantics and Difference scores across clause-level-semantics conditions and age groups. Figure 3 shows the relationship between verb-level semantics (Patient-affectedness ratings) and difference scores across clause-level-semantics conditions (Intentional=Deliberate, Unintentional=Accidental) and age groups (5;6–6;6. 9;6–10;6, Adults).

**Figure 4.**
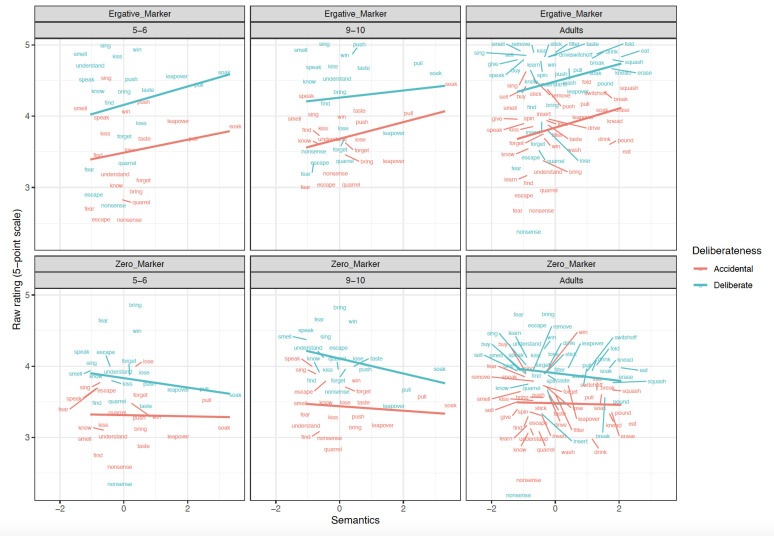
Relationship between verb-level Semantics and Raw scores across clause-level-semantics conditions and age groups. Figure 4 shows the relationship between verb-level semantics (Patient-affectedness ratings) and raw scores across clause-level-semantics conditions (Intentional=Deliberate, Unintentional=Accidental) and age groups (5;6–6;6. 9;6–10;6, Adults).

### Children aged 5;6–6;6

Focussing first on the most informative difference-score analyses ( Table 1), the main effect of **Preemption** ( *p* < .01) was significant in both the simultaneous and single-predictor models. **Intentionality** (clause-level semantics) was a significant predictor in the simultaneous -predictor model ( *p* =0.03) though not the single-predictor models ( *p*=0.06 in both cases; though this would not seem to be a meaningful difference, since the estimate is the same, and the *p* values straddle the 0.05 boundary). This significant effect of Preemption was also observed for raw *ne* marked sentences ( *p* < .05), again in both the simultaneous and single-predictor models, but not for zero-marked sentences. Main effects of Intentionality (clause-level semantics) were also observed for raw ratings of *ne* (as expected) and zero-marked sentences. This latter finding is unexpected, but may reflect a general preference for intentional actions and the unintentional actions being less typical. In summary, the 5–6 year-olds showed clear evidence of Preemption and Intentionality.

### Children aged 9;6–10;6

Although no significant main effects of predictors or interactions were observed for difference-scores ( Table 2), the older children did show the predicted **Intentionality** (clause-level semantics) effect for the raw *ne* marked sentences, in both the single-predictor and simultaneous models. The Preemption effect was nearing significance in the single-predictor model for raw *ne* marked sentences ( *p* < .06). For zero-marked sentences, only a relatively-uninformative main effect of Intentionality was observed (again, presumably reflecting a general preference). In summary, the 5–6 year-olds showed clear evidence of Intentionality though – surprisingly – not preemption.

### Adults

Focussing again on the most-informative difference-score analyses ( Table 3), main effects of Intentionality ( *p* < .01) and Preemption ( *p* < .01) were significant in both the simultaneous and single-predictor models. Unlike for children, a narrowly significant effect of verb-level semantics (i.e., patient-affectedness) was observed ( *p* =0.03) *,* but only in the single-predictor model (though it would have been more comfortably significant if we had pre-registered a one-tailed directional statistical test alongside our directional prediction). The effects of Intentionality (clause-level semantics) and Preemption – but not verb-level semantics – were also observed (again for both simultaneous and single-predictor models) for *ne* marked sentences ( *p values* < .01). For zero-marked sentences, the only significant effects that were observed were unexpected and difficult-to-interpret: a main effect of Intentionality and an interaction of Intentionality x Preemption. In summary, the adults showed clear evidence of Preemption and Intentionality.

### Study 1 (grammatical acceptability judgments): summary

Although the fine-grained pattern of results is rather complicated, overall, a clear picture emerges: All age groups showed clear effects of Intentionality (clause-level semantics) and – apart from the middle age group – Preemption (though this likely reflects a chance finding, rather than genuine U-shaped learning). That is, ergative *ne* marking is preferred when (a) relative to other verbs, the relevant verb is more likely to occur with *ne* than zero-marking in the input and (b) the event is intentional, rather than unintentional. Both of these factors therefore seem to play a key role in learning.

Much more limited evidence was obtained for the effect of verb-level semantics (i.e., patient-affectedness, as determined by the semantic ratings task). However, the fact that such an effect was observed for adults – though only for the single-predictor difference-score model – suggests that, while this factor may play little-to-no role in acquisition *per se* – it may have historically determined which verbs come to prefer *ne* versus zero subject marking.

A potential concern surrounding these conclusions, though, is that they are based entirely on acceptability judgment data, which can be noisy with young children and – even more crucially – may not be directly reflective of the linguistic mechanisms that they use when producing speech (including errors of ergative *ne* marking). We therefore investigated the potential effects of preemption and verb-level semantics in a production study. As discussed earlier, this second study did not investigate intentionality since (a) we were not confident that children could reliably determine intentionality from the animations and (b) we already have clear evidence of a role for intentionality – for all age-groups – from Study 1.

## Study 2: Elicited production

The sample size, methods and data-analysis plan were registered prior to data collection. For the preregistration document, please see *Extended Data* ( Ambridge *et al*., 2023b).

### Participants

48 children were recruited in each age group, 4;0–5;0 ( *M*: 4.70, SD: 0.34) and 5;6–6;6 ( *M*: 6.00, *SD*: 0.32) years, for the elicited production task. Recruitment methods (four schools were approached and all agreed to take part in the study) and ethical approval were same as Study 1. Parents who supervised their children in completing the task received 500INR compensation for their time. We ran a pilot study with seven children aged 4–5 in order to ascertain the feasibility of running an elicited-production task with this age group. These data are not included in the analyses presented below.

### Materials

For the elicited production task, the same list of 40 verbs and animations developed in Study 1 were used. Children watched the animations from the intentional condition only. The animations were accompanied by a “clue word”: the relevant inflected verb form in the simple past tense in both audio and written formats. The verb forms were recorded by a female near-native Hindi speaker on Audacity 2.1.2 ( Audacity Team, 2016). Seven different animations, depicting the actions corresponding to Hindi verbs *jalaana (burn), kaaTnaa (cut), nachaana (dance), (sajaanaa) decorate, (giraanaa) drop, (sukhaana) dry,* and *khiilaana (feed)* were used for the practice trials, which depicted an action involving a boy or an inanimate object.

### Procedure

The experiment ran on the Gorilla platform and children completed the task over Zoom along with a parent or guardian. The experimenter was present during the Zoom session in order to answer any questions that the parents might have when supervising their child but did not conduct the experiment, which was entirely computerized. The task was set up as a game wherein the child watched a video depicting the action denoted by each verb and built a sentence describing the action using the clue word (the verb corresponding to the depicted action, inflected for past tense/perfective inflection) provided by a talking dog. The video was presented first followed by the clue word, and the child's responses audio-recorded. The parent or the guardian was asked to assist the child in audio-recording their responses on the computer, but not to provide any hints regarding the form of the responses themselves. The child was instructed in Hindi on how to complete the task. For the detailed instructions for the task, translated into English, see *Extended data* ( Ambridge *et al*., 2023b). 

All children saw the same list of 40 verbs. The order of presentation of the verbs was fully randomised for each child, using the randomisation function of the Gorilla platform. Each child completed the experiment in a single session which took approximately 30–40 minutes per child. All children completed seven practice trials prior to the main task. In the practice trials, children described the event and received feedback on their responses. The feedback consisted of a correct description of the event which the parent read out to the child. 

### Predictors

The frequency counts (chi-square measure) and patient-affectedness ratings obtained in Study 1 served as the preemption and verb-level semantics predictors for Study 2. For this production study, the preemption account predicts that the higher the (scaled and centred) chi-square value (such that positive and negative values represent a bias towards the *ne* marked and zero marked subjects respectively), the greater the probability of children using the ergative *ne* versus zero marking on the subject (with all other responses discarded as missing data). The verb-level semantics account predicts that the higher the (scaled and centred) patient-affectedness score, the greater the probability of children using the ergative *ne* versus zero marking on the subject.

### Statistical analyses

The data were analysed using R version 3.6.3 (R Studio Version 1.2.5042) ( R Core Team, 2020). All analyses were conducted exactly according to our pre-registered analysis plan which is reproduced below. The preregistered analysis plan was as follows:

The above mentioned hypotheses will be tested in three ways. First, we will run a maximal (as far as will converge) mixed effects model (using lme4 in R) investigating the verb semantics predictor (Hypothesis 2) only - since this is the primary predictor of interest. P values will be calculated via the "drop1" method. Second, we will run a maximal (as far as will converge) mixed effects model (using lme4 in R) investigating the preemption predictor (Hypothesis 1) only. P values will be calculated via the "drop1" method. Third, we will run a maximal (as far as will converge) mixed effects model (using lme4 in R) investigating both the verb semantics and the preemption predictors. P values will be calculated via the "drop1" method (see attached syntax). This is considered a secondary analysis due to likely collinearity between the predictors.

Mixed effects models were run using the lme4 package ( Bates *et al*., 2015); *p* values were obtained using the ‘drop1’ method (i.e., nested models were compared using the likelihood ratio test). Statistical analyses were conducted for each age group separately (4;00–5;00; 5;6–6;6). As in Study 1, we ran both simultaneous and single-predictor models.

### Results

**Figure 5.**
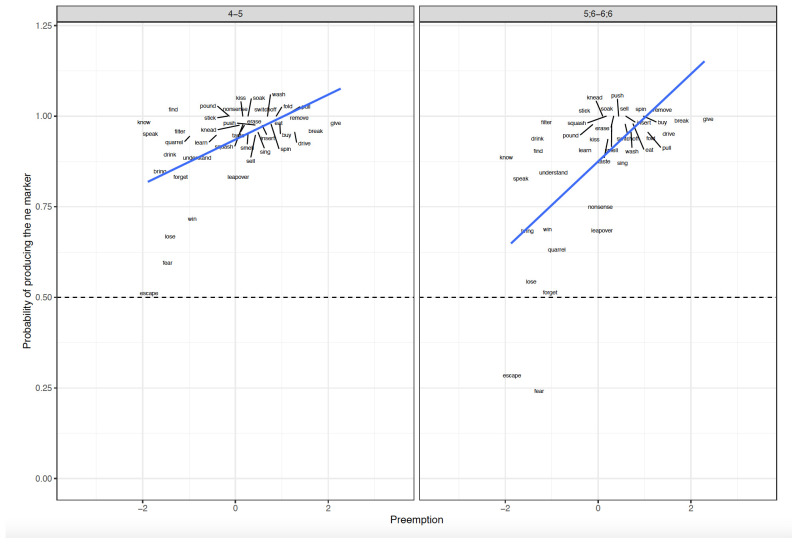
Relationship between Preemption counts and Elicited Production across Age groups. Figure 5 shows the relationship between preemption counts (higher corpus relative verb frequency triggering ne form) and elicited production across age groups (4–5, 5;6–6;6).

**Figure 6.**
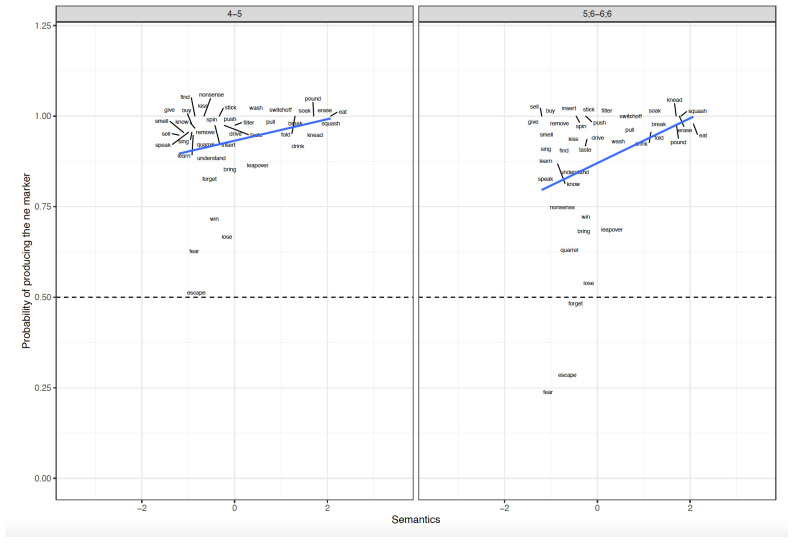
Relationship between verb-level Semantics and Elicited Production across Age groups. Figure 6 shows the relationship between verb-level semantics (Patient Affectedness) and elicited production across age groups (4–5, 5;6–6;6).

For both age groups, main effects of preemption were significant in both the simultaneous and single-predictor models (see Table 10). As expected, the higher the (Z-score standardized) chi-square value operationalizing preemption, the greater the children's production probability of using the ergative marker *ne* on the subject versus zero marking (see Figure 5). Significant effects of Verb Semantics (as measured using the patient affectedness measure) were observed in single-predictor models, but not in the simultaneous models, for both the age groups (see Table 10 & Figure 6). What this suggests is that effects of both preemption and verb semantics are present in the data – and, indeed show broadly similar effect sizes – but that we cannot pick them apart as they are inevitably highly correlated: Verbs that are high in transitivity (Verb level semantics) tend to occur frequently with ergative *ne-* marking in the input (preemption). That said, across both studies, preemption seems to be larger and more robust effect.

**Table 10.** Elicited Production Responses: Simultaneous and Single-predictor Models for Both Age Groups.

	4;0-5;0 year olds				5;6-6;6 year olds			
	*Est*	*SE*	*z*	*p*	*Est*	*SE*	*z*	*p*
Simultaneous								
Intercept	5.23	0.62	8.47	0.00	3.50	0.37	9.48	0.00
** Preemption**	**1.86**	**0.51**	**3.66**	**0.00**	**1.25**	**0.32**	**3.87**	**0.00**
Verb Semantics	-0.38	0.44	-0.86	0.39	0.58	0.38	1.53	0.13
Single-predictor (Preemption)								
Intercept	4.79	0.51	9.39	0.00	3.48	0.37	9.37	0.00
** Preemption**	**1.49**	**0.40**	**3.75**	**0.00**	**1.42**	**0.32**	**4.39**	**0.00**
Single-predictor (Semantics)								
Intercept	4.46	0.47	9.58	0.00	3.58	0.45	7.91	0.00
** Verb Semantics**	**0.91**	**0.36**	**2.50**	**0.01**	**0.91**	**0.45**	**2.05**	**0.04**

### Discussion (Study 2)

 In summary, the results of Study 2 (elicited production) echo those of Study 1 (acceptability judgments) in relation to the effects of preemption which were observed in every analysis. Unlike in Study 1, however, effects of verb level semantics were also observed; though these cannot statistically be teased apart from preemption. Taken together, then Study 1 (acceptability judgments) and Study 2 (elicited production) suggest that statistical preemption is the main mechanism by which children learn which verbs do and do not trigger ergative *ne* marking. However, evidence from Study 1 suggests that clause-level semantics (in the form of intentionality) also influences judgments about the acceptability of ergative *ne* marking. The role of verb-level semantics is less clear: such effects were observed only for adults in Study 1, and only in the single-predictor models for Study 2. Thus, it seems that effects of Verb-level semantics are present in the data, but we cannot tell whether they are actually utilized by children in learning, or have merely historically determined which verbs tend to occur with and without ne- marking in the first place.

## General discussion

 A question that lies at the very heart of language acquisition research is how children learn generalizations with exceptions (e.g., the English plural rule that yields *cats, dogs*, etc, with exceptions *feet* and *men*). Previous research has provided evidence for two accounts. Statistical preemption (e.g., Goldberg, 1995; Goldberg, 2006; Goldberg, 2011; Goldberg, 2019) holds that children are sensitive to the competition between forms with the same (or similar) meanings. For example, in the domain of verb argument structure, repeatedly hearing *[A] made [B]* dance (e.g., *Somebody made the boy dance*) probabilistically outcompetes – that is, statistically pre-empts – *[A] danced [B]* (e.g., *Somebody danced the boy). Semantic accounts hold that learners are guided in part by meaning. For example, the reason we can say *Somebody boiled the water* but not *Somebody danced the boy* is that “boiling” but not “dancing” is an activity that an external causer can more-or-less force another entity to undergo ( Ambridge *et al.*, 2020; Ambridge *et al.*, 2022; Pinker, 1989; Shibatani & Pardeshi, 2002). 

 The present study tested the preemption and semantics hypotheses for Hindi ergative *ne* marking: another semi-regular system characterized by exceptions. The preemption account predicts that the greater the frequency with which a particular verb appears with versus without *ne* marking on the subject – relative to other verbs – the greater the extent to which child and adult participants will (Study 1) accept and (Study 2) produce *ne* over zero-marked subjects. The semantics hypothesis was tested at both the verb and the clause level: At the verb level, this account predicts that the greater the verb’s semantic transitivity (as determined in a separate semantic-rating task of patient affectedness) the greater the extent to which child and adult participants will (Study 1) accept and (Study 2) produce *ne* over zero-marked subjects. At the clause level, this account predicts that when an action is portrayed as intentional rather than unintentional, child and adult participants will show a greater preference (Study 1) for *ne* over zero-marked subjects.

 Overall, the findings from the acceptability-judgment study (with 5–6 year-olds, 9–10 year-olds and adults) and the production study (with 4–5 and 5–6 year olds) yield a clear picture. Findings of statistical preemption were observed across the board, suggesting that the main way Hindi-speaking children learn which verbs do and do not trigger *ne* marking is probabilistic input-based learning at the verb level. At the same time, the acceptability-judgment study shows that learners also seem to be sensitive to a clause-level semantic constraint such that intentional actions require *ne* marking to a greater extent than unintentional ones. Effects of verb-level semantics, however, were observed only sporadically, primarily in the adult acceptability judgment data and elicited production data from children. These findings indicate that to some extent, *ne* marking is associated with higher transitivity actions, but this effect disappears when controlling for preemption. Most likely then, transitivity (verb-level semantics) determines which verbs prefer *ne*- versus zero marking historically, but learners primarily learn these patterns statistically on a verb-by-verb basis (i.e., via statistical preemption).

Before turning to the theoretical implications, it is important to acknowledge that the present studies constitute only a first step towards investigating the complex semantic factors that influence *ne*- versus zero marking. As noted earlier, amongst all of the potentially-relevant semantic factors set out by Hopper and Thomspon (1980), amonst others, we investigate only the “degree of affectedness of the patient” (via the semantic rating task) and “conscious activity of the agent” (via the intentionality manipulation). Futhermore, as pointed out by a reviewer, Aaricia Ponnet, the present studies do not consider intentionality differences that are inherent to the meaning of the verb (e.g., *see* vs *look at* vs *watch* in English), nor – since the study was restricted to two-participant transitive verbs – how they affect *ne*- versus zero marking of single-participant transitive verbs (e.g., *dance* vs *sneeze*). These factors should be investigated in future research with both adults and children.

In the meantime, the present results therefore add to a growing body of work which suggests that learners acquire exception-filled generalizations by (a) learning probabilistically from the input which surface form is used by adult speakers to convey a particular meaning (i.e., via statistical preemption) and (b) forming overarching generalizations based on semantics, which allow them to generalize to new scenarios. For Hindi ergative *ne* marking, these semantic effects seem mainly to operate at the level of the clause, specifically reflecting intentionality. That is, in terms of avoiding overgeneralizations, children learn that the exceptions to ergative *ne-* marking occur in contexts with low levels of intentionality. The role of verb-level semantic effects warrants further investigation. Future studies investigating other language systems characterized by partial productivity should therefore take seriously, and investigate empirically, the possibility that effects of semantics exist. In the meantime, the present findings have demonstrated that, for a previously understudied phenomenon – ergative *ne-* marking – learners acquire exception-filled generalizations using both verb-by-verb learning (i.e., statistical preemption) and forming overarching semantic generalizations (here, intentionality).

## Ethics and consent

The study was approved by ethics committees at the University of Liverpool, UK (RETH001041) and International Institute of Information Technology – Hyderabad, India (IIITH-IRB-PRO-2021-02). Written informed consent was obtained using online and physical consent forms, depending on whether the task was completed face-to-face or online. For children, written informed consent was obtained from parents. Children provided verbal assent.

## Figures and Tables

**Table:** Appendix

Hindi_Sentences_Deliberate		Hindi_Sentences_ Accidental		English_ Sentences_ Deliberate	English_ Sentences_ Accidental
ne marked	zero marked	ne marked	zero marked		
लडके ने दूध चखा।	लडका दूध चखा।	लडके ने गलती से दूध चखा।	लडका गलती से दूध चखा।	The boy tasted the milk	The boy accidentally tasted the milk
लडके ने कपड़ा भिगोया।	लडका कपड़ा भिगोया।	लडके ने गलती से कपड़ा भिगोया।	लडका गलती से कपड़ा भिगोया।	The boy soaked the cloth	The boy accidentally soaked the cloth
लडके ने ढक्कन हटाया।	लडका ढक्कन हटाया।	लडके ने गलती से ढक्कन हटाया।	लडका गलती से ढक्कन हटाया।	The boy removed the lid	The boy accidentally removed the lid
लडके ने केक खाया।	लडका केक खाया।	लडके ने गलती से केक खाया।	लडका गलती से केक खाया।	The boy ate the cake	The boy accidentally ate the cake
लडके ने खिलौना चूमा।	लडका खिलौना चूमा।	लडके ने गलती से खिलौना चूमा।	लडका गलती से खिलौना चूमा।	The boy kissed the toy	The boy accidentally kissed the toy
लडके ने तार खींचा।	लडका तार खींचा।	लडके ने गलती से तार खींचा।	लडका गलती से तार खींचा।	The boy pulled the wire	The boy accidentally pulled the wire
लडके ने बाक्स धकेला।	लडका बाक्स धकेला।	लडके ने गलती से बाक्स धकेला।	लडका गलती से बाक्स धकेला।	The boy pushed the box	The boy accidentally pushed the box
लडके ने कैन पिचकाया।	लडका कैन पिचकाया।	लडके ने गलती से कैन पिचकाया।	लडका गलती से कैन पिचकाया।	The boy squashed the can	The boy accidentally squashed the can
लडके ने गाना सीखा।	लडका गाना सीखा।	लडके ने गलती से गाना सीखा।	लडका गलती से गाना सीखा।	The boy learnt the song	The boy accidentally learnt the song
लडके ने कप धोया।	लडका कप धोया।	लडके ने गलती से कप धोया।	लडका गलती से कप धोया।	The boy washed the cup	The boy accidentally washed the cup
लडके ने फूल मिटाया।	लडका फूल मिटाया।	लडके ने गलती से फूल मिटाया।	लडका गलती से फूल मिटाया।	The boy erased the flower	The boy accidentally erased the flower
लडके ने पानी छाना।	लडका पानी छाना।	लडके ने गलती से पानी छाना।	लडका गलती से पानी छाना।	The boy filtered the water	The boy accidentally filtered the water
लडके ने खिलौना खरीदा।	लडका खिलौना खरीदा।	लडके ने गलती से ट्रक खरीदा।	लडका गलती से ट्रक खरीदा।	The boy bought the toy	The boy accidentally bought the truck
लडके ने लट्टू घुमाया।	लडका लट्टू घुमाया।	लडके ने गलती से लट्टू घुमाया।	लडका गलती से लट्टू घुमाया।	The boy spun the spinning top	The boy accidentally spun the spinning top
लडके ने बल्ब बुझाया।	लडका बल्ब बुझाया।	लडके ने गलती से बल्ब बुझाया।	लडका गलती से बल्ब बुझाया।	The boy switched off the bulb	The boy accidentally switched off the bulb
लडके ने पानी पिया।	लडका पानी पिया।	लडके ने गलती से पानी पिया।	लडका गलती से पानी पिया।	The boy drank the water	The boy accidentally drank the water
लडके ने खिलौना दिया।	लडका खिलौना दिया।	लडके ने गलती से खिलौना दिया।	लडका गलती से खिलौना दिया।	The boy gave the toy	The boy accidentally gave the toy
लडके ने खिलौना बेचा।	लडका खिलौना बेचा।	लडके ने गलती से खिलौना बेचा।	लडका गलती से खिलौना बेचा।	The boy sold the toy	The boy accidentally sold the toy
लडके ने चित्र चिपकाया।	लडका चित्र चिपकाया।	लडके ने गलती से चित्र चिपकाया।	लडका गलती से चित्र चिपकाया।	The boy stuck the painting	The boy accidentally stuck the painting
लडके ने आटा गूंधा।	लडका आटा गूंधा।	लडके ने गलती से आटा गूंधा।	लडका गलती से आटा गूंधा।	The boy kneaded the dough	The boy accidentally kneaded the dough
लडके ने मसाला कूटा।	लडका मसाला कूटा।	लडके ने गलती से मसाला कूटा।	लडका गलती से मसाला कूटा।	The boy pounded the spices	The boy accidentally pounded the spices
लडके ने स्कूटर चलाया।	लडका स्कूटर चलाया।	लडके ने गलती से स्कूटर चलाया।	लडका गलती से स्कूटर चलाया।	The boy drove the scooter	The boy accidentally drove the scooter
लडके ने कागज मोडा।	लडका कागज मोडा।	लडके ने गलती से कागज मोडा।	लडका गलती से कागज मोडा।	The boy folded the paper	The boy accidentally folded the paper
लडके ने ट्रक तोडा।	लडका ट्रक तोडा।	लडके ने गलती से ट्रक तोडा।	लडका गलती से ट्रक तोडा।	The boy broke the truck	The boy accidentally broke the truck
लडके ने कलम घुसाया।	लडका कलम घुसाया।	लडके ने गलती से कलम घुसाया।	लडका गलती से कलम घुसाया।	The boy inserted the pen	The boy accidentally inserted the pen
लडके ने राक्षस से डरा।	लडका राक्षस से डरा।	लडके ने गलती से राक्षस से डरा।	लडका गलती से राक्षस से डरा।	The boy feared (from) the monster	The boy accidentally feared (from) the monster
लडके ने स्वेटर पर झगडा।	लडका स्वेटर पर झगडा।	लडके ने गलती से स्वेटर पर झगडा।	लडका गलती से स्वेटर पर झगडा।	The boy quarrelled for the sweater	The boy accidentally quarrelled for the sweater
लडके ने बुक लाया।	लडका बुक लाया।	लडके ने गलती से बुक लाया।	लडका गलती से बुक लाया।	The boy brought the newspaper	The boy accidentally brought the newspaper
लडके ने हीट से बचा।	लडका हीट से बचा।	लडके ने गलती से हीट से बचा।	लडका गलती से हीट से बचा।	The boy escaped (from) the heat	The boy accidentally escaped (from) the heat
लडके ने उत्तर भूला।	लडका उत्तर भूला।	लडके ने गलती से उत्तर भूला।	लडका गलती से उत्तर भूला।	The boy forgot the answer	The boy accidentally forgot the answer
लडके ने भाषण समझा।	लडका भाषण समझा।	लडके ने गलती से भाषण समझा।	लडका गलती से भाषण समझा।	The boy understood the speech	The boy accidentally understood the speech
लडके ने गाना गाया।	लडका गाना गाया।	लडके ने गलती से गाना गाया।	लडका गलती से गाना गाया।	The boy sang the song	The boy accidentally sang the song
लडके ने खेल जीता।	लडका खेल जीता।	लडके ने गलती से खेल जीता।	लडका गलती से खेल जीता।	The boy won the game	The boy accidentally won the game
लडके ने बाड़ फांदा।	लडका बाड़ फांदा।	लडके ने गलती से बाड़ फांदा।	लडका गलती से बाड़ फांदा।	The boy leaped over the fence	The boy accidentally leaped over the fence
लडके ने उत्तर बका।	लडका उत्तर बका।	लडके ने गलती से उत्तर बका।	लडका गलती से उत्तर बका।	The boy talked nonsense answer	The boy accidentally talked nonsense answer
लडके ने खेल हारा।	लडका खेल हारा।	लडके ने गलती से खेल हारा।	लडका गलती से खेल हारा।	The boy lost the game	The boy accidentally lost the game
लडके ने कलम पाया।	लडका कलम पाया।	लडके ने गलती से कलम पाया।	लडका गलती से कलम पाया।	The boy found the pen	The boy accidentally found the pen
लडके ने उत्तर जाना।	लडका उत्तर जाना।	लडके ने गलती से उत्तर जाना।	लडका गलती से उत्तर जाना।	The boy knew the answer	The boy accidentally knew the answer
लडके ने फूल सूंघा।	लडका फूल सूंघा।	लडके ने गलती से फूल सूंघा।	लडका गलती से फूल सूंघा।	The boy smelt the flower	The boy accidentally smelt the flower
लडके ने समाचार बोला।	लडका समाचार बोला।	लडके ने गलती से समाचार बोला।	लडका गलती से समाचार बोला।	The boy spoke the news	The boy accidentally spoke the news

## Data Availability

OSF: Ergative marking in Hindi: Stimuli, data and R code https://doi.org/10.17605/OSF.IO/7KS63 ( Ambridge *et al*., 2023a). This project contains the following underlying data: File name.pdf/xlsx/mp4 (brief description of file (in a few words)) ErgativeStudy_Animations.zip (contains animations for the grammatical judgment and elicited production tasks in intentional (e.g., BREAK.mp4) and unintentional (e.g., BREAK_A.mp4) conditions; only animations in the intentional condition were used for the elicited production task) Ergative_Judgment_SentencesAudio.zip (contains audio files that accompanied the animations in the main trial of Study 1) ErgativeStudy_PracticeAnimations.zip Ergative_Judgment_PracticeAnimations (contains animations and audio files that accompanied the animations in the practice trial of Study 1) Ergative_Production_PracticeAnimations (contains animations and audio files that accompanied the animations in the practice trial of Study 2) ErgativeStudy_VerbLists_Practice&Main.zip Ergative_Hindi_VerbLists.xlsx (contains three sub-sheets which have details on the verbs that were used for adults and children - one adult list; two child lists - Child_List1, Child_List2) ErgativeGJ_PracticeTrialsList.xlsx (contains list of the trials used for the grammatical judgment practice task in Study 1) Ergative_EP_PracticeTrialsList.xlsx (contains list of the trials used for the elicited production practice task in Study 1) Ergative_SemanticsTaskMaterials.zip (contains materials used in the semantics ratings task) ErgativeStudy_Data&RCode-ORE.zip Judgment_Study_Data (contains data and R code for Study 1) finalrawdata-wp2-GJ-5-6-nopreemp.csv (raw data for 5–6 year olds) finalrawdata-wp2-GJ-9-10-nopreemp.csv (raw data for 9–10 year olds) finalrawdata-wp2-GJ-adults-nopreemp.csv (raw data for adults) PreemptionCounts.csv (contains the frequency counts for all 40 verbs) raw-semantics-20.csv (contains the raw data obtained from 20 participants on the semantics ratings task) semantics-20-mean scores.xlsx (contains the mean ratings across participants for each verb on the semantics ratings task) V2_Ergative_Judgments.R (R code for Study 1) Production_Study_Data (contains data and R code for Study 2) Production_FinalCodeSheet_4–5 (contains raw data from 4–5 year olds) Production_FinalCodeSheet_5–6 (contains raw data from 5–6 year olds) PreemptionCounts.csv (contains the frequency counts for all 40 verbs) raw-semantics-20.csv (contains the raw data obtained from 20 participants on the semantics ratings task) semantics-20-mean scores.xlsx (contains the mean ratings across participants for each verb on the semantics ratings task) Ergative_Prod.R (R code for Study 2) OSF: CLASS: Cross Linguistic Acquisition of Sentence Structure https://doi.org/10.17605/OSF.IO/PAVM7 ( Ambridge *et al*., 2023b). This project contains the following extended data: File name.pdf/xlsx/mp4 (brief description of file (in a few words)) Pre-registration document for Study 1 (Ambridge, B., Maitreyee, R., Narasimhan, B., Sharma, D. M., Nair, R. B., & Samanta, S. (2019). Development of ergative case-marking in Hindi: Evidence from a grammaticality judgment study (preregistration). *Open Science Framework*. https://doi.org/10.17605/OSF.IO/Q5RT8 Pre-registration document for Study 2 (Maitreyee, R., Ambridge, B., Narasimhan, B., Saxena, G., Sharma, D. M., & Nair, R. B. (2019). The roles of preemption and semantics in the production of ergative marking in Hindi speaking children (preregistration). *Open Science Framework*. https://doi.org/10.17605/OSF.IO/H678K Data are available under the terms of the Creative Commons Attribution 4.0 International license (CC-BY 4.0).

## Footnotes

^1^ Also, the verb agrees with the highest null-marked argument in the clause and if all arguments receive overt case, the verb exhibits masculine singular default agreement Mohanan (1994) in order to maintain uniformity in verb agreement, the present study used masculine singular nouns for the subject and masculine nouns for the object arguments of transitive verbs.

^2^ The age data for 2 children were missing because of technical faults, so the mean age was calculated from the data obtained for 46 children.

^3^ There was one exception, i.e. for the verb ‘fear from’, we used an animate noun (‘demon’) as the patient.

^4^ In fact, the model syntax in the preregistration contained a typo such that the Verb-semantics*Intentionality interaction was not included, although it is clear from the main text of the pre-registration that it was intended to be. Thus the model did include this analysis.
