# Supplementary material for: Children learn ergative case marking in Hindi using statistical preemption and clause-level semantics (intentionality): evidence from acceptability judgment and elicited production studies with children and adults
Source: Open Res Eur. 2023 Sep 13;3:49. Originally published 2023 Mar 29. [Version 2] doi: 10.12688/openreseurope.15611.2 (PMC10466009; doi:10.12688/openreseurope.15611.2)
Supplement: Peer review history

# Reviewer report: Reviewer response for version 1

**DOI:** 10.21956/openreseurope.16872.r33459

**Published:** 2023-08-29

**Authors:** Aaricia Ponnet

**Affiliations:** 1 Linguistics, Ghent University, Pietersnieuwstraat, Gent, Belgium

This article adds some fundamental findings to ongoing research about how children learn certain verbal constructions and how they deal with exceptions and semantic restrictions when learning verbal event constructions. Moreover, they do this by focussing on Hindi, a language which is, despite being the third most spoken language in the world, still under-researched in linguistic research and especially in research with a language acquisition and learnability perspective. Moreover, the study focuses on child speakers of Hindi in India of two different age groups (with a younger pilot study group) as well as a group of adult speakers. This means that the study not only provides interesting data from a learnability perspective (i.e. how do children deal with learning the different semantic and syntactic restrictions that are related to the acquisition of the construction under study) but also adds to the growing body of work on the Hindi language and additionally gives valuable insights on the distribution of *ne* marking with adult speakers of Hindi in India. It taps into to the body of work that researches the use and acquisition of differential case marking with children and adults, in this case, split ergativity (with a focus on active, transitive, two-participant verbs).

 One of the strong points of this study is that it not only provides a wide range of data with considerably large sample groups with more than 140 participants in total (given that this type of research in India is still rather novel, and given the restrictions during the COVID pandemic during which this research has been performed, this is quite an impressive number), but it also provides a solid hypothesis and contrasts two different linguistic theoretical approaches that the study aims to test by analysing the data of the different speaker groups. On the one hand, the study aims to predict and test ‘statistical preemption’ (based on recent work by Goldberg and collaborators) which poses that certain constructions or linguistic forms probabilistically outcompete others in the input distribution of children, which means that they will prefer certain constructions or forms over others. On the other, the study aims to elaborate on previous work on the role of semantics in the learnability of certain constructions (building on previous work by Ambridge and collaborators), and typological work on Hindi (a.o. Mohanan 1994) that highlights the effects of verb-level semantics, in the case of split ergativity: transitivity, and clause level semantics, in the case of split ergativity: volitionality or intentionality. Verbs that rate higher on the transitivity cline or that are associated with a higher sense of intentionality are predicted to be associated with *ne* with a higher range of frequency. It is important to note that the study focuses on active, transitive, two-participant verbs – intransitive verbs that can in some cases take the ergative were not included.

 Data were collected using an acceptability judgment task (three groups, children of different age groups and adults) and an elicited production task (two groups, a younger group and a group aged 5-6). These are presented as two separate studies (study 1 and study 2), after which the results are compared in the discussion section. Study 1 looks at the effect of statistical preemption, intentionality and transitivity. Study 2 investigates the effect of statistical preemption and transitivity. A large online Hindi corpus was perused to account for statistical preemption with regard to the quantitative analysis. Another strong feature of the study is that it openly shares the material that was used to collect the data online, as well as the analyses in R. This means that the study is replicable, but also that reviewers and readers can have all the necessary information needed to interpret and check the results of this study.

 Both the studies (acceptability judgment and elicited production) find an effect of statistical preemption over the different age groups (except for the middle age group in Study 1). In Study 1, additionally, a significant effect for intentionality was attested, but not for transitivity. In Study 2, to a lesser extent, an effect was found also for transitivity. This leads the authors to the conclusion that children rely both on frequency as well as semantics to learn certain regularities as well as exceptions to these regularities. In the case of ergative *ne *marking then, and specifically with regard to transitive perfective verbs, children learn, according to the authors, on a verb-by-verb basis, and with those verbs where *ne* marking is optional in particular, are further guided by semantics, in this case, intentionality. As per my knowledge, this is the first study on Hindi ergative marking that has been carried out at such a large scale, which makes it a necessary, innovative contribution to the field of linguistics.

 The study is in my opinion a valuable and perhaps even groundbreaking contribution with regard to the several aspects that I summarized and highlighted above. I do have, however, some minor reservations/questions regarding the following:

- A list of the 40 verbs is given in an online repository, but for the interpretation of the results of the study, I find it essential to include at least an overview of the verbs in the article itself (preferably with the original Hindi verb as well as its English counterpart).
- In Hindi, the subject is generally null-marked (nominative). What makes the split ergative construction in Hindi challenging is that there is an opposition between ergative *ne *marking and nominative *null* marking. Children have to learn that the opposition transitive/intransitive is morphologically marked with verbs that have the perfective aspect. The verbs that have been included in this study only concern verbs that can have ergative marking but not typical intransitive verbs such as *jaanaa* ‘to go’, *bhaagnaa* ‘to run’, *baiThnaa* ‘to sit’, *khaaMsnaa* ‘to cough’, *naacnaa* ‘to dance’ and so on, neither does it include such intransitive verbs that can sometimes have subject marked with the ergative such as *chiikh* ‘to scream’. On the one hand, this focus on two-participant transitive verbs could be made clearer in the introduction of the study (and, consequently, the exclusion of single-participant intransitive verbs), on the other hand, the inclusion of intransitive perfective verbs is in my opinion elementary to the research of the learnability of split ergativity as a whole, not only the semantics that are involved but also the morphosyntactic opposition between ergative and nominative. This is particularly so since there are certain intransitive verbs that allow for *ne *marking when volitionality/intentionality is at play – and these verbs were not included in the study. Moreover, when testing statistical preemption, there will also be a role of the frequency of certain lemmas in the subject function – there can only be *ne *marking of the subject in the perfective aspect verb form, all other aspects have nominative subjects. Since other verb forms were not included in the study, the effect of frequency of nominative on the subject forms is therefore not accounted for in this study. Especially from a language development perspective, the question remains how children shift from mainly nominative to mainly ergative – if such a shift is present at all. This aspect is not covered by the study as most of the age groups perform similarly to the adult group, which suggests that by age 4-5 these nuances are already acquired.
- Another issue regards the rating of the affectedness of the patient to rate the level of transitivity, by which the authors aim to test the influence of verb-level semantics on the use of *ne* with active, transitive two-participant verbs. This was tested by presenting participants passivized forms of the 40 verbs that were included in the test. My concern is that the effect of transitivity cannot be entirely tested in this way. Moreover, with regard to the transitivity cline, there are several aspects that have been stated by Hopper & Thompson (1980) that influence the level of transitivity, which concerns the agent as well as the patient. Moreover, according to Hopper & Thompson (1980), transitivity is “a global property of an entire clause” (251). Maitreyee et al. distinguish between intentionality at the clause level and transitivity as verb-level semantics, while these features may very well both be positioned at the clause level. Either the choice for this distinction at the semantic level needs to be made more clear by the authors in the text, or the focus should be more on ‘patient affectedness’ and less on transitivity, as transitivity encompasses more than affectedness alone. Moreover, volitionality/agentivity (which highly overlaps with the feature ‘intentionality’ that is being investigated in this study) is one of the features stated by Hopper and Thompson that influences the rate of transitivity as well (252). Within this line of reasoning, the authors have researched the effect of two transitivity features on the use of *ne* (i.e. intentionality vs. patient affectedness) rather than intentionality vs. transitivity.
- Intentionality was tested by presenting the participants with four sentences, alternately marked with *ne* or null-marked in the nominative, and by adding an adverb *galtii se* ‘by mistake’. In this way sentences were created that were, according to the authors, more intentional than others within the meaning of that particular verb. However, 1) an adverb expressing higher intentionality was not added, and 2) the range of intentionality between the different verbs that were tested was not accounted for. The figures in the article show a clear difference between sentences that have the adverb ‘by mistake’ and verbs that have not, which confirms a successful operationalization of ‘less intentional’, but there is also a cline to be observed between the verbs at the same level of intentionality. This probably goes beyond the scope of the study but confirms that differences in intentionality are inherent to the verbs and not solely attributable to the addition of adverbs. Moreover, actions with intransitive verbs can also be highly intentional (consider, e.g., to laugh and constructions such as laughing with vs. laughing at) and intentionality thus inevitably also interacts with the discourse-pragmatic level. I would have expected the authors to mention this briefly in the discussion section of the study.
- I believe these data and analyses are extremely valuable and I hope that for future research, the researchers are considering adding a qualitative component as well. From a language development perspective, I would have loved to see a few cases and example sentences highlighted that give us more insight into the details of the acquisition and use of ergative marking with Hindi-speaking children. This evidently goes beyond the scope of the study, but a qualitative analysis will give us more information on the between-verb differences in use at the same level as what the authors operationalised as intentionality and transitivity, as well as interindividual differences, between the different age groups as well as between the different participants within the same age group. Is there a learning curve or have all these children indeed acquired the semantic and syntactic constraints? Are some verbs more difficult than others? What were the highest or clearest contrasts in language use in the use of ergative marking within the adult groups, and what does that tell us about the regularity vs. optionality of *ne* marking, vs. the different features that interact with *ne *marking? I applaud the authors for creating the possibility of answering such questions with their collected data and am excited to read more of their work in the future.

Please find an annotated pdf of the article with further comments here.

If applicable, is the statistical analysis and its interpretation appropriate?

I cannot comment. A qualified statistician is required.

Is the study design appropriate and is the work technically sound?

Yes

Is the work clearly and accurately presented and does it engage with the current literature?

Yes

Are the conclusions drawn adequately supported by the results?

Yes

Are sufficient details of methods and analysis provided to allow replication by others?

Yes

Are all the source data and materials underlying the results available?

Yes

Reviewer Expertise:

Second language acquisition, Hindi, General Linguistics, Morphosyntax, Syntax-semantics interface, differential case marking

I confirm that I have read this submission and believe that I have an appropriate level of expertise to confirm that it is of an acceptable scientific standard, however I have significant reservations, as outlined above.

# Response

**Published:** 2023-09-01

**Authors:** Benjamin AMBRIDGE

We thank Dr Ponnet for this very detailed and thoughtful review. Below we summarize the changes we have made in response (highlighted with ++).

 “The study is in my opinion a valuable and perhaps even groundbreaking contribution with regard to the several aspects that I summarized and highlighted above. I do have, however, some minor reservations/questions regarding the following: A list of the 40 verbs is given in an online repository, but for the interpretation of the results of the study, I find it essential to include at least an overview of the verbs in the article itself (preferably with the original Hindi verb as well as its English counterpart)”.

 +Thanks for this very useful suggestion: We have now added an appendix with the full list of sentences

 “In Hindi, the subject is generally null-marked (nominative). What makes the split ergative construction in Hindi challenging is that there is an opposition between ergative ne marking and nominative null marking. Children have to learn that the opposition transitive/intransitive is morphologically marked with verbs that have the perfective aspect. The verbs that have been included in this study only concern verbs that can have ergative marking but not typical intransitive verbs such as jaanaa ‘to go’, bhaagnaa ‘to run’, baiThnaa ‘to sit’, khaaMsnaa ‘to cough’, naacnaa ‘to dance’ and so on, neither does it include such intransitive verbs that can sometimes have subject marked with the ergative such as chiikh ‘to scream’. On the one hand, this focus on two-participant transitive verbs could be made clearer in the introduction of the study (and, consequently, the exclusion of single-participant intransitive verbs), on the other hand, the inclusion of intransitive perfective verbs is in my opinion elementary to the research of the learnability of split ergativity as a whole, not only the semantics that are involved but also the morphosyntactic opposition between ergative and nominative. This is particularly so since there are certain intransitive verbs that allow for ne marking when volitionality/intentionality is at play – and these verbs were not included in the study. Moreover, when testing statistical preemption, there will also be a role of the frequency of certain lemmas in the subject function – there can only be ne marking of the subject in the perfective aspect verb form, all other aspects have nominative subjects. Since other verb forms were not included in the study, the effect of frequency of nominative on the subject forms is therefore not accounted for in this study. Especially from a language development perspective, the question remains how children shift from mainly nominative to mainly ergative – if such a shift is present at all. This aspect is not covered by the study as most of the age groups perform similarly to the adult group, which suggests that by age 4-5 these nuances are already acquired”.

 ++These are all important points and we agree completely, but – short of adding additional studies – we can see no way to address them in the present paper other than to acknowledge and highlight these omissions, and highlight them for future research. We have therefore added the following passage: “Indeed, as a reviewer, Aaricia Ponnet, pointed out, by restricting the present study to two-participant transitive verbs, we have already simplified the task facing learners: Although ergative case-marking is predominantly found with two-participant verbs, it can also optionally occur on the single argument of a small set of intransitive verbs (e.g., chiikh ‘scream’), apparently marking a volitional action (e.g., Butt & King, 1991; Butt & King, 2003; de Hoop & Narasimhan, 2005; Mohanan, 1994). And, of course, as Ponnet highlights, learning this “morphosyntactic opposition between ergative and nominative” – as it applies across both intransitive and transitive verbs – is “elementary…to the learnability of split ergativity as a whole”.  Future studies, then, should include this full range of verbs.

 Focussing on the present set of transitive verbs, these considerations raise the possibility that semantic effects on Hindi ergative ne marking, if they exist at all, may occur not at the verb level, but at the clausal level…”

 “Another issue regards the rating of the affectedness of the patient to rate the level of transitivity, by which the authors aim to test the influence of verb-level semantics on the use of ne with active, transitive two-participant verbs. This was tested by presenting participants passivized forms of the 40 verbs that were included in the test. My concern is that the effect of transitivity cannot be entirely tested in this way. Moreover, with regard to the transitivity cline, there are several aspects that have been stated by Hopper & Thompson (1980) that influence the level of transitivity, which concerns the agent as well as the patient. Moreover, according to Hopper & Thompson (1980), transitivity is “a global property of an entire clause” (251). Maitreyee et al. distinguish between intentionality at the clause level and transitivity as verb-level semantics, while these features may very well both be positioned at the clause level. Either the choice for this distinction at the semantic level needs to be made more clear by the authors in the text, or the focus should be more on ‘patient affectedness’ and less on transitivity, as transitivity encompasses more than affectedness alone. Moreover, volitionality/agentivity (which highly overlaps with the feature ‘intentionality’ that is being investigated in this study) is one of the features stated by Hopper and Thompson that influences the rate of transitivity as well (252). Within this line of reasoning, the authors have researched the effect of two transitivity features on the use of ne (i.e. intentionality vs. patient affectedness) rather than intentionality vs. transitivity”.

 ++As for the previous comment, this is again a valid and important point, but one that cannot be satisfactorily addressed without adding new studies/manipulations. Again, then, we simply acknowledge the omission, and highlight it as something for future research, adding the following: “It is important to acknowledge and highlight the fact that, as pointed out by a reviewer, Aaricia Ponnet, this semantic rating task measures just one aspect of potentially-relevant semantics: patient affectedness. Theoretical analyses of transitivity highlight its multifactorial nature, with transitivity affected not only by the “degree of affectedness of the patient”, but also “the effectiveness with which an action takes place, the punctuality and telicity of the verb, the conscious activity of the agent, [and] the referentiality…of the object” (e.g., Hopper & Thompson, 1980: 251). Although our additional intentionality manipulation investigates the effect of “conscious activity of the agent” (as we note in the Discussion, only to a limited extent), future studies should address the other semantic factors that are likely to be relevant to learning ergative -ne versus zero marking”.

 “Intentionality was tested by presenting the participants with four sentences, alternately marked with ne or null-marked in the nominative, and by adding an adverb galtii se ‘by mistake’. In this way sentences were created that were, according to the authors, more intentional than others within the meaning of that particular verb. However, 1) an adverb expressing higher intentionality was not added, and 2) the range of intentionality between the different verbs that were tested was not accounted for. The figures in the article show a clear difference between sentences that have the adverb ‘by mistake’ and verbs that have not, which confirms a successful operationalization of ‘less intentional’, but there is also a cline to be observed between the verbs at the same level of intentionality. This probably goes beyond the scope of the study but confirms that differences in intentionality are inherent to the verbs and not solely attributable to the addition of adverbs. Moreover, actions with intransitive verbs can also be highly intentional (consider, e.g., to laugh and constructions such as laughing with vs. laughing at) and intentionality thus inevitably also interacts with the discourse-pragmatic level. I would have expected the authors to mention this briefly in the discussion section of the study”.

 ++Again, this is entirely correct and, as the reviewer suggests, we have now mentioned this issue briefly in the discussion section as follows: “Before turning to the theoretical implications, it is important to acknowledge that the present studies constitute only a first step towards investigating the complex semantic factors that influence ne- versus zero marking. As noted earlier, amongst all of the potentially-relevant semantic factors set out by Hopper and Thomspon (1980), amonst others, we investigate only the “degree of affectedness of the patient” (via the semantic rating task) and “conscious activity of the agent” (via the intentionality manipulation). Futhermore, as pointed out by a reviewer, Aaricia Ponnet, the present studies do not consider intentionality differences that are inherent to the meaning of the verb (e.g., see vs look at vs watch in English), nor – since the study was restricted to two-participant transitive verbs – how they affect ne- versus zero marking of single-participant transitive verbs (e.g., dance vs sneeze). These factors should be investigated in future research with both adults and children”.

 “I believe these data and analyses are extremely valuable and I hope that for future research, the researchers are considering adding a qualitative component as well. From a language development perspective, I would have loved to see a few cases and example sentences highlighted that give us more insight into the details of the acquisition and use of ergative marking with Hindi-speaking children. This evidently goes beyond the scope of the study, but a qualitative analysis will give us more information on the between-verb differences in use at the same level as what the authors operationalised as intentionality and transitivity, as well as interindividual differences, between the different age groups as well as between the different participants within the same age group. Is there a learning curve or have all these children indeed acquired the semantic and syntactic constraints? Are some verbs more difficult than others? What were the highest or clearest contrasts in language use in the use of ergative marking within the adult groups, and what does that tell us about the regularity vs. optionality of ne marking, vs. the different features that interact with ne marking? I applaud the authors for creating the possibility of answering such questions with their collected data and am excited to read more of their work in the future”.

 ++We thank Dr Ponnet for these generous comments. Although, as the review notes, such qualitative analysis is generally beyond the scope of the study, we have added a brief section that discusses the qualitative patterns observed in the data; touching briefly on the issues of between-verb differences/clearest constrasts for adults, differences between the child age groups, regularity/optionality of ne/zero marking, and the different features (verb/clause level semantics) that interact with it. The added section is as follows: “Qualitative analyses Before turning to the preregistered statistical analyses, it is informative to first briefly discuss the qualitative patterns observed (see Figures 1-4), particularly with regard to the verb- and clause-level semantic manipulations, seen most clearly in Figure 3 (for discussion and analysis of Hindi children’s acquisition of ergative case marking “in the wild” see Narasimhan, 2005). Looking first at the adult data (rightmost panel of Figure 3), one of the most striking findings is that while only a handful of verbs prefer zero marking, we see a smooth cline from verbs that prefer -ne marking very strongly (e.g., break, filter, smell, spin, push) to those that prefer -ne over zero marking only marginally, particularly when the action is non-intentional (e.g., speak, understand, lose, talk nonsense). Also intriguing is the pattern shown by verbs such as win and lose, which prefer ne- marking for intentional actions, but flip to a preference for zero marking for unintentional actions. This suggests that ergative marking is multifactorial and probabilistic, and cannot be captured by a straightforward division of verbs into those that trigger ne-/zero/optional marking. The child data are similar to the adult data in that both child groups seem to show broadly-adultlike effects of both patient-affectedness and intentionality (particularly for zero-preferring verbs such as fear, escape, quarrel and forget), though with a few exceptions. For example, both child groups overestimate – relative to adults – -ne preference for soak; the older children particularly so for unintentional soaking.”

 ++We again end by thanking Dr Ponnet for this enthusiastic, detailed and thoughtful review.

# Reviewer report: Reviewer response for version 1

**DOI:** 10.21956/openreseurope.16872.r33451

**Published:** 2023-07-26

**Authors:** Vsevolod Kapatsinski

**Affiliations:** 1 Department of Linguistics, University of Oregon, Eugene, OR, USA

This paper addresses the long-standing question of how speakers learn lexical restrictions on construction use in a new (to this literature) language, Hindi. It argues that the choice of the ergative particle 'ne' is constrained by both semantic transitivity of the sentence and the specific verb, with the verbs that most often occur with 'ne' being judged as less acceptable without it. This latter effect is said to provide evidence for statistical preemption. 6-year-olds, 10-year-olds and adults are tested. The effects of preemption / lexical idiosyncrasy are stronger than those of semantics.

 The paper makes a valuable contribution to the literature by extending this debate to a new language, and a new construction. There is also methodological innovation in developing a way to rate semantic transitivity. Another strength of the paper is that both judgments and production data are collected.

 One aspect of the paper that is not clear to me is whether it actually tests preemption. The tested prediction is "the greater the frequency with which a particular verb appears with versus without *ne* marking on the subject – relative to other verbs in the test set – the greater the extent to which *ne* marked forms will be preferred over zero-marked forms in the judgment task... we operationalized this measure using a scaled and centred chi-square statistic which measures how often, in past tense/perfective aspect active sentences, a particular verb triggers ergative case marking as compared with all other verbs in the test set of 40". However, this conflates frequency in the rated construction (e.g., with ne) and frequency in the other construction (without ne). The latter is an implementation of preemption but the former is not. How do we know that frequency in the other construction matters for the judgments? One way to address this issue would be to include frequency in the presented construction as a separate predictor. Less of an issue, but this also does not necessarily differentiate preemption and entrenchment, the frequency with which the verb occurs in other constructions that are not  synonymous with the 'ne'. 

 A limitation of the analyses is that no interactions between age/subject group and the other predictors are fitted. These would show whether the effects of preemption and semantics actually change significantly over the tested age span.

If applicable, is the statistical analysis and its interpretation appropriate?

Yes

Is the study design appropriate and is the work technically sound?

Yes

Is the work clearly and accurately presented and does it engage with the current literature?

Yes

Are the conclusions drawn adequately supported by the results?

Partly

Are sufficient details of methods and analysis provided to allow replication by others?

Yes

Are all the source data and materials underlying the results available?

Yes

Reviewer Expertise:

Learning theory, usage-based linguistics

I confirm that I have read this submission and believe that I have an appropriate level of expertise to confirm that it is of an acceptable scientific standard, however I have significant reservations, as outlined above.

# Response

**Published:** 2023-08-29

**Authors:** Benjamin AMBRIDGE

We thank Prof Kapatsinski for raising these two important points. With regard to the first, we have added the following passage: A reviewer, Vsevolod Kapatsinski, suggested that this chi-square measure “conflates frequency in the rated construction (e.g., with ne) and frequency in the other construction (without ne). The latter is an implementation of preemption but the former is not. How do we know that frequency in the other construction matters for the judgments?”. We accept that because preemption was originally presented as a descriptive verbal theory (i.e., not an equation or computational model) there is room for disagreement about precisely how it should be instantiated numerically. However, we maintain that any valid measure of preemption must reflect the relative corpus frequency of the form that occurred (here, a ne-marked noun) and a form that feasibly could have but did not occur (here, a zero-marked noun). This is clear in, for example, the following definition from Goldberg (2011), with Hindi examples added in square brackets:

 In a situation in which a construction A (CxA) might have been expected to be uttered with a given verb, verb i, the learner can infer that CxA [e.g., laRke=ne, ‘boy-Erg’] is not after all appropriate for that verb, if a different construction, CxB [e.g., laRkaa-0, ‘boy-Nom’], is consistently witnessed instead.

 Indeed, a version of preemption (or learning/reasoning more generally) that did not take into account the “base rate” in this way would be very difficult to support on logical grounds; like arguing that Drug A (5 deaths amongst 50 patients) is safer than Drug B (10 deaths amongst 1,000 patients). For this reason, we follow previous studies that have used the chi-square/Fisher’s exact method for calculating the measure of preemption/competition (e.g., Stefanowitsch, 2008; Ambridge et al., 2018; Tatsumi, Ambridge & Pine, 2018a, 2018b; Ambridge et al., 2020; Ambridge et al, 2022; Liu & Ambridge, 2020; Ambridge, Arnon & Bekman, 2023). With regard to the second point, we carefully considered adding the interactions with age but decided not to because it would constitute a significant departure from our pre-registered analysis plan, particularly given that - since we did not plan to analyse the interactions - we did not power our study to detect them. This means that any failure to find interactions would be difficult to interpret, and could well reflect nothing more than a lack of power. Furthermore, the reason that we did not include the interactions in the preregistered analysis plan is that it is unclear both (a) whether the theories under investigation would predict any change in the magnitude of the effects (particularly given that adults complete a somewhat different version of the study to children) and (b) whether any apparent change could be distinguished from task/group effects. Indeed, the descriptive finding from Study 1 - that effects of preemption and intentionality "disappear" between age 5-6 and 9-10 only to reappear in adults, can only really feasibly be task/group effects.

# Reviewer report: Reviewer response for version 1

**DOI:** 10.21956/openreseurope.16872.r33450

**Published:** 2023-07-26

**Authors:** Archna Bhatia

**Affiliations:** 1 Florida Institute for Human and Machine Cognition, Ocala, Florida, USA

This article explores how children learn semi-regular systems (exceptions-filled generalizations) in languages through an example of ergative case marking in Hindi which shows irregularity in that it appears in sentences with transitive verbs in perfective aspect and in sentences with some intransitive verbs that involve volitionality but not with intransitive verbs that lack volitionality also in perfective aspect, also they may be optional in sentences with intransitive verbs. Authors are interested in testing whether such systems are learnt as probabilistic generalizations or as formal rules with memorized exceptions (semantically restricted generalizations). Authors conduct a grammaticality judgement task and an elicited production task to test the probabilistic generalization hypothesis and the semantically restricted generalization hypothesis for learning semi-regular systems.

 For the probabilistic generalization hypothesis, they study whether the ne-marked forms’ frequency compared to zero-marked forms’ frequency with a particular verb has an impact on its acceptability or production. To test the hypothesis about learning guided by semantic rules, e.g., only verbs high on transitivity trigger ergative ne-marking, they study the extent of ne- vs zero-marked subjects appearing with verbs rated higher on transitivity as being acceptable or produced. To test the semantics hypothesis at the verb-level, transitivity is operationalized in terms of the degree to which the patient is affected by the action denoted by the verb. To test the semantics hypothesis at the clause-level, authors study the impact of an action appearing intentional rather than unintentional on the extent to which ne-marking forms compared to zero-marked forms are rated as acceptable.

 Authors recruited children in age groups 5-6 and 9-10 and adults in their studies. The studies’ results indicated that learners acquire exceptions-filled generalizations by learning probabilistically based on the input as well as forming overarching semantic generalizations and learning semantics based exceptions to avoid overgeneralization. The probabilistic generalization (statistical preemption) effects were observed across the board.  In terms of semantics, authors observed that learners were sensitive to a clause level semantic constraint involving intentionality of the actions. Authors found limited evidence for the effect of verb-level semantics (patient affectedness), however for adults this effect was observed indicating potential historical links between patient-affectedness and whether the verb would come to prefer ne vs zero marking.

 The work is presented in the paper clearly. The study design is appropriate, and sufficient details are provided about the study design as well as methods and analyses used for the studies in general. The work is technically sound. Authors have made the data they prepared and used in their studies publicly available which will make the replication of the work possible as well as provide data for further analyses and for use in further studies. In my understanding, the statistical analyses and the interpretations seem appropriate, however a statistician may provide further comments. The conclusions are well-supported.

 Regarding the 40 selected action verbs, did authors look at the inter-annotator agreement between the two native speakers providing intuitions about the action verbs whether they appeared with ergative ne-marking zero-marking or optional ne-marking?

If applicable, is the statistical analysis and its interpretation appropriate?

Yes

Is the study design appropriate and is the work technically sound?

Yes

Is the work clearly and accurately presented and does it engage with the current literature?

Yes

Are the conclusions drawn adequately supported by the results?

Yes

Are sufficient details of methods and analysis provided to allow replication by others?

Yes

Are all the source data and materials underlying the results available?

Yes

Reviewer Expertise:

Speech and Natural Language Processing, Linguistics

I confirm that I have read this submission and believe that I have an appropriate level of expertise to confirm that it is of an acceptable scientific standard.

# Response

**Published:** 2023-08-29

**Authors:** Benjamin AMBRIDGE

This reviewer raises only one issue: "Regarding the 40 selected action verbs, did authors look at the inter-annotator agreement between the two native speakers providing intuitions about the action verbs whether they appeared with ergative ne-marking zero-marking or optional ne-marking?" We did not do this because our native-speaker classifications into ne/zero/optional verbs were informal, and used only to select a range of verbs; they were not used as a factor in any statistical analysis. It is only the ratings of our participants that reveal the "true" degree of grammaticality of ne vs zero marked forms.
